# Cardiac autonomic function score: a novel risk stratification tool in the cardiac intensive care unit based on periodic repolarization dynamics and deceleration capacity of heart rate (LMU-eICU study)

**DOI:** 10.1093/ehjdh/ztaf038

**Published:** 2025-04-30

**Authors:** Mathias Klemm, Lukas von Stülpnagel, Valentin Ostermaier, Carsten Theurer, Laura E Villegas Sierra, Felix Wenner, Elodie Eiffener, Aresa Krasniqi, Konstantinos Mourouzis, Lauren E Sams, Luisa Freyer, Steffen Massberg, Axel Bauer, Konstantinos D Rizas

**Affiliations:** Medizinische Klinik und Poliklinik I, LMU Klinikum, Ludwigs-Maximilians-University Munich, Ziemsenstr. 5, 80336 Munich, Germany; German Centre for Cardiovascular Research (DZHK), Partner Site Munich Heart Alliance, Pettekoferstr. 9, 80336 Munich, Germany; Medizinische Klinik und Poliklinik I, LMU Klinikum, Ludwigs-Maximilians-University Munich, Ziemsenstr. 5, 80336 Munich, Germany; German Centre for Cardiovascular Research (DZHK), Partner Site Munich Heart Alliance, Pettekoferstr. 9, 80336 Munich, Germany; University Hospital for Internal Medicine III, Medical University of Innsbruck, Anichstr. 35, 6020 Innsbruck, Austria; Medizinische Klinik und Poliklinik I, LMU Klinikum, Ludwigs-Maximilians-University Munich, Ziemsenstr. 5, 80336 Munich, Germany; Medizinische Klinik und Poliklinik I, LMU Klinikum, Ludwigs-Maximilians-University Munich, Ziemsenstr. 5, 80336 Munich, Germany; Medizinische Klinik und Poliklinik I, LMU Klinikum, Ludwigs-Maximilians-University Munich, Ziemsenstr. 5, 80336 Munich, Germany; German Centre for Cardiovascular Research (DZHK), Partner Site Munich Heart Alliance, Pettekoferstr. 9, 80336 Munich, Germany; Medizinische Klinik und Poliklinik I, LMU Klinikum, Ludwigs-Maximilians-University Munich, Ziemsenstr. 5, 80336 Munich, Germany; German Centre for Cardiovascular Research (DZHK), Partner Site Munich Heart Alliance, Pettekoferstr. 9, 80336 Munich, Germany; Medizinische Klinik und Poliklinik I, LMU Klinikum, Ludwigs-Maximilians-University Munich, Ziemsenstr. 5, 80336 Munich, Germany; German Centre for Cardiovascular Research (DZHK), Partner Site Munich Heart Alliance, Pettekoferstr. 9, 80336 Munich, Germany; Medizinische Klinik und Poliklinik I, LMU Klinikum, Ludwigs-Maximilians-University Munich, Ziemsenstr. 5, 80336 Munich, Germany; German Centre for Cardiovascular Research (DZHK), Partner Site Munich Heart Alliance, Pettekoferstr. 9, 80336 Munich, Germany; Medizinische Klinik und Poliklinik I, LMU Klinikum, Ludwigs-Maximilians-University Munich, Ziemsenstr. 5, 80336 Munich, Germany; German Centre for Cardiovascular Research (DZHK), Partner Site Munich Heart Alliance, Pettekoferstr. 9, 80336 Munich, Germany; Medizinische Klinik und Poliklinik I, LMU Klinikum, Ludwigs-Maximilians-University Munich, Ziemsenstr. 5, 80336 Munich, Germany; German Centre for Cardiovascular Research (DZHK), Partner Site Munich Heart Alliance, Pettekoferstr. 9, 80336 Munich, Germany; Medizinische Klinik und Poliklinik I, LMU Klinikum, Ludwigs-Maximilians-University Munich, Ziemsenstr. 5, 80336 Munich, Germany; German Centre for Cardiovascular Research (DZHK), Partner Site Munich Heart Alliance, Pettekoferstr. 9, 80336 Munich, Germany; Medizinische Klinik und Poliklinik I, LMU Klinikum, Ludwigs-Maximilians-University Munich, Ziemsenstr. 5, 80336 Munich, Germany; German Centre for Cardiovascular Research (DZHK), Partner Site Munich Heart Alliance, Pettekoferstr. 9, 80336 Munich, Germany; University Hospital for Internal Medicine III, Medical University of Innsbruck, Anichstr. 35, 6020 Innsbruck, Austria; Medizinische Klinik und Poliklinik I, LMU Klinikum, Ludwigs-Maximilians-University Munich, Ziemsenstr. 5, 80336 Munich, Germany; German Centre for Cardiovascular Research (DZHK), Partner Site Munich Heart Alliance, Pettekoferstr. 9, 80336 Munich, Germany

**Keywords:** Intensive care unit, Autonomic function, Risk stratification, Periodic repolarization dynamics, Deceleration capacity of heart rate

## Abstract

**Aims:**

Treatment capacities on intensive care units (ICUs) are a limited resource reserved for high-risk patients. To facilitate risk stratification of ICU patients, several scoring systems have been developed over time. Among them, the Simplified Acute Physiology Score 3 (SAPS3) is the gold standard, but lacks specificity for cardiac ICU patients. Here, we propose a novel, fully automated, electrocardiogram-based cardiac autonomic risk stratification score (CAF_ICU_) that substantially adds to current risk stratification strategies.

**Methods and results:**

CAF_ICU_ is based on periodic repolarization dynamics, a marker of sympathetic overactivity and deceleration capacity of heart rate, a parameter of vagal imbalance. We developed CAF_ICU_ in a retrospective cohort of 355 ICU patients and subsequently validated the score in a cohort of 702 ICU patients, enrolled between February–November 2018 and December 2018–April 2020 at a large cardiac ICU in a tertiary hospital. The primary endpoint of the study was 30-day intrahospital mortality. Thirty (8.5%) and 100 (14.2%) patients reached the primary endpoint in the training and validation cohorts, respectively. CAF_ICU_ was significantly higher in non-survivors than survivors (2.58 ± 1.34 vs. 1.76 ± 0.97 units; *P* = 0.003 in the training cohort and 2.20 ± 1.05 vs. 1.70 ± 0.83 units; *P* < 0.001 in the validation cohort) and was a strong predictor of mortality in both the training [hazard ratio (HR) 25.67; 95% confidence interval (CI) 3.50–188.40; *P* = 0.001] and validation cohorts (HR 4.70; 95% CI 2.79–7.92; *P* < 0.001). In the pooled cohort, CAF_ICU_ significantly improved risk stratification based on SAPS3 (IDI-increase 0.033; 95% CI 0.010–0.061; *P* < 0.001).

**Conclusion:**

ECG-based automatic autonomic risk stratification by means of PRD and DC is highly predictive of short-term mortality in the ICU and can be combined with the SAPS3-Score to identify patients with increased risk for intrahospital mortality. This method can be integrated in conventional monitors and may improve risk stratification strategies in cardiac ICUs.

## Structured Graphical Abstract

**Structured Graphical Abstract:**
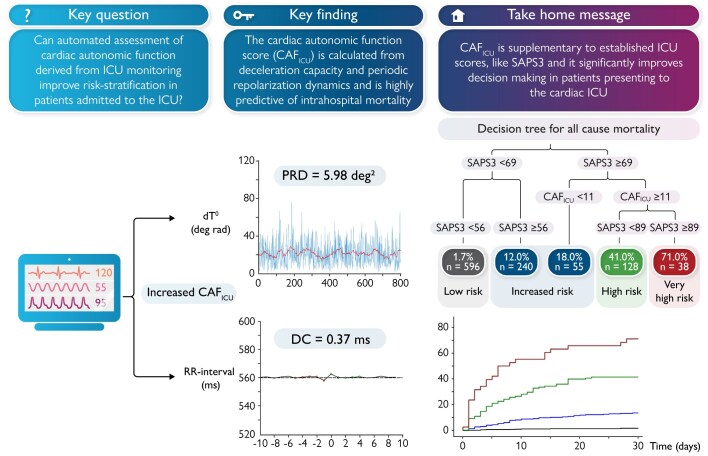

## Introduction

Treatment capacities in intensive care units (ICUs) are a critical resource in modern medicine. For medical professionals working in ICU, accurate triaging algorithms are therefore of high importance. To facilitate risk stratification of ICU patients, several scoring systems have been developed over time, including the Sepsis-related Organ Failure Assessment (SOFA) score, the Acute Physiology and Chronic Health Evaluation II (APACHE II) score, the Simplified Acute Physiology Score 3 (SAPS3), and the Mortality Probability Admission Model (MPM).^1–4^ All these scores were developed at least 20 years ago in non-cardiac ICUs and therefore are not optimized for cardiac patients treated with modern therapies. Moreover, while some of these scores include the patient’s heart rate as risk variable, they do not consider the state of the patient’s autonomic nervous system (ANS).

The ANS is an integrated neural network connecting all vital organ systems. Damage to an organ within this network has an impact on the functional status of the ANS. The status of the ANS can be assessed non-invasively by analysing biosignals derived from electrocardiogram (ECG) recordings.^5^ As ECG monitoring is standard in any patient treated in the ICU, integration of continuous ANS assessment in ICU risk stratification scores is feasible and could lead to improved risk stratification algorithms. This would not only facilitate correct triaging of low-risk patients, but could also lead to prompt identification of patients at high risk for cardiovascular complications, such as cardiogenic shock, who might benefit from invasive treatments, including the use of extracorporeal life support (ECLS).

Periodic repolarization dynamics (PRD)^6–8^ and deceleration capacity of heart rate (DC)^9,10^ are advanced digital ANS biomarkers, which specifically assess the function of the sympathetic and parasympathetic nervous systems, respectively. Both markers have been shown to be strong predictors of mortality, cardiovascular mortality, and sudden cardiac death (SCD) in patients suffering from various cardiovascular diseases.^6–10^ However, so far, these variables have not been used to predict risk when assessed from continuous ECG monitors in the ICU.

In this study, we aimed to develop a novel cardiac autonomic function score (CAF_ICU_) based on PRD and DC, using ECGs recorded from patient monitors during the first night of ICU stay. The study was divided into a training and validation phase. We hypothesized that the new ANS score would correctly identify high- as well as low-risk patients in the ICU and would improve established ICU risk stratification algorithms.

## Methods

### Study design

Patients admitted to the cardiac ICU at the university hospital of Munich/Campus Grosshadern from 1 February 2018 to 30 April 2020 were retrospectively screened for eligibility. The inclusion criteria were the presence of sinus rhythm and the availability of an ECG recording, derived from patient monitoring system during the first night of ICU stay. The exclusion criteria were atrial fibrillation or other arrhythmia and non-availability of ECG data and/or patient records. For patients with more than one ICU stay, only the initial stay was considered. The study was divided into two phases: (i) training phase, between 1 February 2018 and 30 November 2018 and (ii) validation phase, between 1 December 2018 and 30 April 2020. In a *post hoc* sensitivity analysis, we identified patients presenting with atrial fibrillation (AF) during both phases of the study. Both phases of the study were approved by the medical Ethics Committee of the LMU Hospital (18-724 and 21-1270).

### Wave data acquisition and analysis

Patient monitoring at the cardiac ICU of the LMU Hospital/Campus Grosshadern was carried out by means of the Philips IntelliVue MX800 monitoring system. Data acquired from point-of-care patient monitoring were archived in a ‘Microsoft SQL Server’-based database. Electrocardiogram recordings were acquired at a sampling frequency of 250 Hz. The stored ECG recordings were converted to an open-source format (physionet, MIT-format) and automatically annotated using a previously described algorithm.^11^ To ensure standardized conditions for each patient, we extracted and analysed recordings obtained within the first night of the ICU stay between 2:00 a.m. and 2:30 a.m. Deceleration capacity of heart rate, which is an integral measure of all deceleration-related oscillations of heart rate and therefore an indirect quantifier of vagal activity, was calculated according to previously published algorithms. The first step was the calculation of the RR intervals for all ECG recordings. The signals were checked for the presence of AF using a validated automated algorithm.^12^ The RR time series were processed using the phase-rectified signal averaging (PRSA) algorithm, which identifies instances within the RR-interval time series, where the heart rate decelerates (anchors). The central part of the PRSA signal was quantified by Haar wavelet analysis to obtain an estimate of DC.^9^ As described for noisy signals derived from patient monitors, a low-pass filter of *T* = 4 for anchor-point selection was used.^13,14^ The algorithm for calculating PRD has been previously published.^6–8^ All ECG recordings were converted to orthogonal Frank leads and subsequently to a vectorcardiogram, defined by two angles (azimuth and elevation) and the amplitude of the resultant vector ($\sqrt{X^{2\;}+\;Y^{2}\;+\;Z^{2}}$). The weight-averaged direction of repolarization (*T°*) was constructed for all T waves within the observation period, defined by the weight-averaged azimuth and the weight-averaged elevation. In a third step, the instantaneous degree of repolarization instability was estimated by means of the angle *dT°*, which is defined as the scalar product of two successive repolarization vectors *T°* (Equation 3 in Rizas *et al*.^8^). Periodic repolarization dynamics was quantified from the *dT°* signal after adaptation of continuous wavelet transformation and calculation of the average wavelet coefficient, corresponding to frequencies of 0.1 Hz or less. Besides DC and PRD, the following parameters of heart rate variability (HRV) were considered: very low (VLF), low (LF), and high (HF)-frequency band of HRV, as well as the ratio between LF and HF, HRV index (HRVi), the root mean square of successive differences between normal heartbeats (RMSSD), and standard deviation of normal RR intervals (SDNN). All these parameters were calculated from the frequency and time domain, according to the recommendations provided by the task force.^5^ In a *post hoc* sensitivity analysis, we calculated PRD in patients presenting with AF. All calculations were performed using, a previously validated, open-source, MATLAB-based software (SMARTlab; v. 1.5.4).^15^

### Clinical data

Clinical data were retrospectively acquired from the patients’ files. The Simplified Acute Physiology Score 3 was calculated using the following information:^2^ age, length as well as location of intrahospital stay before ICU admission, use of vasoactive drugs before ICU admission, planned or unplanned ICU admission, as well as reason for ICU admission, in case of a surgery the anatomical site and surgical status at ICU admission, Glasgow Coma Scale, acute infection, total bilirubin, body temperature, creatinine, heart rate, pH, leucocytes and platelet count, systolic blood pressure, oxygenation, and other comorbidities. Additional risk factors including intubation, previous CPR, use of ECLS, and haemodialysis were considered. Cardiogenic shock was classified according to the clinical expert consensus statement of the Society for Cardiovascular Angiography and Interventions (SCAI score).^16^

### Study endpoints

The primary endpoint of the study was time to intrahospital mortality. All patients were censored at a maximum follow-up time of 30 days after ICU admission. Patients discharged within 30 days were assumed alive at Day 30 after ICU admission. The secondary endpoint was defined as the composite of intrahospital mortality, ventricular fibrillation (VF), and sustained ventricular tachycardia (VT).

### Sample size calculation

The study population was divided into training and validation cohorts. For the training cohort, the minimum required sample size was calculated using the events per variable criterion,^17^ which is defined by the ratio of the number of events relative to the number of degrees of freedom (parameters) included in the multivariable model. Assuming 3 degrees of freedom for the tested co-variates (PRD, DC, and SAPS3), at least 30 events were required for the study to be adequately powered. Taking into consideration a 30-day mortality rate of 10%, a sample size of at least 300 patients was calculated for the training cohort. The validation cohort was planned with the intention to include at least the double number of patients compared with the training cohort, which would provide enough power for subgroup analyses and model discrimination. For these analyses, both cohorts were merged into a pooled cohort.

### Statistical analysis

Continuous variables are presented as medians with interquartile ranges (IQRs) and were compared using the Wilcoxon rank-sum test. Categorical variables are expressed as absolute numbers and percentages and were compared using the *χ*^2^ test. Multivariable analyses were implemented by the adaptation of Cox regression analysis. The beta coefficients for PRD and DC derived from Cox regression models in the training cohort were calculated. CAF_ICU_ was subsequently defined as the cumulative risk derived from both increasing PRD (beta_PRD_ × PRD) and decreasing DC (beta_DC_ × DC); (CAF_ICU_ = round [10 × exp(beta_PRD_ × PRD + beta_DC_ × DC)])}. For patients, in whom PRD was not available, because of non-availability of three ECG leads, we used the corresponding exponential coefficients derived from univariable Cox regression models, which only included DC. The DC values ≥ 4.5 were considered normal. Periodic repolarization dynamics was capped at the maximum value of 30 deg^2^, DC at the minimum value of 0 ms, and CAF_ICU_ at the minimum value of 0 and maximum value of 100 units. The cut-off value of CAF_ICU_ was defined as the median in the training cohort. The predictive value of CAF_ICU_ was validated in the validation cohort. Survival curves and cumulative proportions were estimated using the Kaplan–Meier method with 95% CI calculated based on Greenwood’s method and were compared using log-rank statistics. The sensitivity and specificity of CAF_ICU_ were analysed using the area under the receiver operating characteristics (ROC) curve and Youden’s *J* statistic. The incremental prognostic value of CAF_ICU_ on top of SAPS3 was tested in the pooled cohort using integrated discrimination improvement index (IDI), continuous net reclassification index (NRI), and median improvement score.^18^ Decision trees for classification of patients based on the SAPS3 and CAF_ICU_ scores were implemented in the pooled cohort by adoption of recursive partitioning and regression trees.^19^ Subgroup analyses for the primary endpoint were performed in the pooled cohort by adjusting the multivariable models for the interaction term between CAF_ICU_ and the corresponding subgroup identiﬁer. The seasonal effect on mortality, resuscitation, sepsis, and intubation was tested using sliding windows with width = 60 days and step size of 10 days. The current month was derived from the median day of the window. A propensity-score-matched sensitivity analysis was performed to adjust for differences in baseline characteristics between included and excluded patients. The baseline characteristics that significantly differed between included and excluded patients were identified, and the probability for a patient to be excluded from the study was calculated. Patients were classified into subclasses and the corresponding weights based on the subclass membership were computed. These weights were used as propensity score weights and applied to estimate the weighted treatment effect in Cox regression analysis.^20^ For this analysis, missing values were replaced using a bootstrapping-based imputation analysis.^21^ For all other calculations, patients with missing values were excluded from the analyses. Differences were considered statistically significant at an alpha level of 0.05. All statistical analyses were performed using R (version 4.3.1) and adoption of the following packages: survival (version 3.5.5), survIDINRI (version 1.1.2), rpart (version 4.1.21), ggplot2 (version 3.4.2), and survminer (version 0.4.9).

## Results

**Figure 1:**
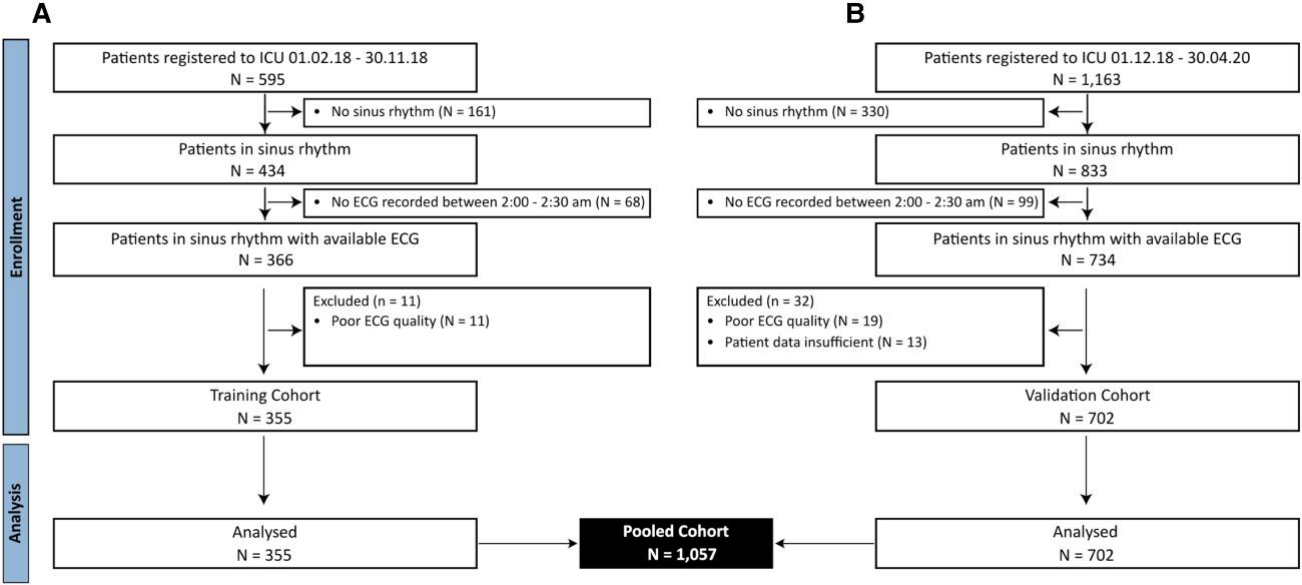
CONSORT diagram for the training (*A*) and validation cohort (*B*). CONSORT, Consolidated Standards of Reporting Trials; ECG, electrocardiogram.

During the enrolment period of the training cohort, 595 unique patients were registered in the cardiac ICU at the university hospital of Munich. Among them, 355 patients had sufficient ECGs recorded in the first night and were in sinus rhythm. The main reasons for exclusion were rhythms other than sinus rhythm (*n* = 161) and missing or insufficient wave data (*n* = 79; *Figure 1A*). The median age was 67 (54–78) years; 125 (35.2%) patients were females. The main reason for admission was acute coronary syndrome (ACS) (49%). One hundred and twenty-three (34.6%) patients required mechanical or medical circulatory support, 79 (22.3%) patients were intubated, 57 (16.1%) received cardiopulmonary resuscitation (CPR) before admission to the ICU, and 92 (25.9%) patients presented with SCAI stage ≥ C. Thirty patients (8.5%) died within 30 days after ICU admission.

During the enrolment period of the validation cohort, 1163 unique patients were registered in the cardiac ICU. Among them, 330 patients were not in sinus rhythm; 137 patients were excluded because of missing or insufficient ECG recordings or unavailable patient data (*Figure 1B*). The median age was 70 (58–80) years, 233 (33.2%) patients were females, 265 (37.7%) required mechanical or medical circulatory support, 162 (23.1%) were intubated, 72 (10.3%) received CPR prior to ICU admission, and 104 (14.8%) patients presented with SCAI stage ≥ C. Out of the 702 patients included into the analysis, 100 (14.2%) died within 30 days after ICU admission.

Overall, the baseline characteristics in the training and validation cohorts were comparable (*Table 1* and Supplementary material online, *Table S1*). However, patients in the validation cohort were older (70 ± 22 vs. 67 ± 24 years, *P* = 0.003), had a lower rate of ACS (32.2 vs. 49.0%, *P* < 0.001), higher rate of aortic valve replacement (18.4 vs. 10.1%, *P* < 0.001), were less frequently smokers (36.2 vs. 43.9%, *P* = 0.002), and more frequently required treatment with haemodialysis (11.1 vs. 3.1%, *P* < 0.001). The median SAPS3 (53.0 ± 19.0 in the validation vs. 52.0 ± 22.0 in the training, *P* = 0.602) was balanced between the two cohorts.

**Table 1:** Baseline characteristics in the training and validation cohorts

	Training cohort				Validation cohort			
	Total (*n* = 355)	Dead (*n* = 30)	Alive (*n* = 325)	*P*-value	Total (*n* = 702)	Dead (*n* = 100)	Alive (*n* = 602)	*P*-value
Age, years (SD)	67 (24)	72 (19)	66 (24)	0.354	70 (22)	70 (16)	71 (23)	0.275
BMI, kg/m^2^ (SD)	26.0 (6.0)	25.5 (7.0)	26.0 (7.0)	0.170	26.1 (5.8)	26.3 (4.7)	26.0 (5.8)	0.532
Female sex (%)	125 (35.2%)	9 (30%)	116 (35.7%)	0.671	233 (33.2%)	32 (32.0%)	201 (33.3%)	0.874
ACS (%)	174 (49%)	9 (30%)	165 (50.8%)	0.044	226 (32.2%)	32 (32.0%)	194 (32.2%)	1.000
ADHF (%)	45 (12.7%)	6 (20%)	39 (12%)	0.338	108 (15.4%)	21 (21.0%)	87 (14.5%)	0.129
AVR (%)	36 (10.1%)	1 (3.3%)	35 (10.8%)	0.330	129 (18.4%)	6 (6.0%)	123 (20.4%)	<0.001
MVR/TVR (%)	11 (3.1%)	0 (0%)	11 (3.4%)	0.636	26 (3.7%)	1 (1.0%)	25 (4.2%)	0.208
Sepsis (%)	24 (6.8%)	7 (23.3%)	17 (5.2%)	<0.001	59 (8.4%)	25 (25.0%)	34 (5.6%)	<0.001
Circulatory support (%)	123 (34.6%)	25 (83.3%)	98 (30.2%)	<0.001	265 (37.7%)	85 (85.0%)	180 (29.9%)	<0.001
Catecholamines (%)	121 (34.1%)	25 (83.3%)	96 (29.5%)	<0.001	263 (37.5%)	85 (85.0%)	178 (29.6%)	<0.001
ECLS (%)	23 (6.5%)	5 (16.7%)	18 (5.5%)	0.048	51 (7.3%)	29 (29.0%)	22 (3.7%)	<0.001
Hypertension (%)	238 (67%)	15 (50%)	223 (68.6%)	0.461	472 (67.2%)	51 (51.0%)	421 (69.9%)	<0.001
CAD (%)	256 (72.1%)	20 (66.7%)	236 (72.6%)	0.797	461 (65.7%)	59 (59.0%)	402 (66.8%)	0.163
CPR prior to admission (%)	57 (16.1%)	16 (53.3%)	41 (12.6%)	<0.001	72 (10.3%)	36 (36.0%)	36 (6.0%)	<0.001
Intubation (%)	79 (22.3%)	18 (60%)	61 (18.8%)	<0.001	162 (23.1%)	71 (71.0%)	91 (15.1%)	<0.001
Diabetes (%)	85 (23.9%)	9 (30%)	76 (23.4%)	0.303	201 (28.6%)	32 (32.0%)	169 (28.1%)	0.500
Smokers (%)	156 (43.9%)	9 (30%)	147 (45.2%)	0.380	254 (36.2%)	23 (23.0%)	231 (38.4%)	0.004
COPD (%)	40 (11.3%)	4 (13.3%)	36 (11.1%)	0.739	62 (8.8%)	10 (10.0%)	52 (8.6%)	0.807
Dialysis (%)	11 (3.1%)	3 (10%)	8 (2.5%)	0.058	78 (11.1%)	37 (37.0%)	41 (6.8%)	<0.001
Max creatinine within 24 h, mg/dL (IQR)	1.2 (0.7)	1.7 (1.6)	1.1 (0.7)	<0.001	1.1 (0.7)	1.7 (1.3)	1.1 (0.6)	<0.001
SCAI A	221 (63.3%)	6 (20.0%)	215 (66.2%)	<0.001	487 (69.4%)	32 (32.0%)	455 (75.6%)	<0.001
SCAI B	34 (9.6%)	3 (10.0%)	31 (9.5%)	1.000	88 (12.5%)	10 (10.0%)	78 (13.0%)	0.664
SCAI C	10 (2.8%)	1 (3.3%)	9 (2.8%)	1.000	12 (1.7%)	2 (2.0%)	10 (1.7%)	1.000
SCAI D	59 (16.6%)	11 (36.7%)	48 (14.8%)	<0.001	41 (5.8%)	18 (18.0%)	23 (3.8%)	<0.001
SCAI E	23 (6.5%)	5 (16.7%)	18 (5.5%)	0.023	51 (7.3%)	29 (29.0%)	22 (3.7%)	<0.001
DC, ms (IQR)	3.9 (5.2)	1.6 (3.2)	4.1 (5.3)	< 0.001	3.8 (4.7)	1.5 (2.3)	4.2 (4.6)	< 0.001
PRD, deg^2^ (IQR)	7.7 (8.6)	12.9 (7.0)	7.2 (8.7)	0.004	7.9 (8.3)	9.3 (8.8)	7.8 (8.1)	0.031
SAPS3 score (IQR)	52.0 (24)	75.0 (25.5)	51.0 (24.0)	<0.001	53.0 (19.0)	74.5 (15.2)	51.0 (15.7)	<0.001
CAF_ICU_ (IQR)	11 (7)	14 (8)	11 (6)	<0.001	11 (5)	13 (6)	10 (6)	<0.001

ACS, acute coronary syndrome; ADHF, acute decompensated heart failure; AVR, aortic valve replacement; BMI, body mass index; CAD, coronary artery disease; CAF_ICU_, cardiac autonomic function score in the ICU; CPR, cardiopulmonary resuscitation; DC, deceleration capacity of heart rate; ECLS, extracorporeal circulatory life support; MVR, mitral valve repair; PRD, periodic repolarization dynamics; SAPS3, Simplified Acute Physiology Score 3; SCAI, Society for Cardiovascular Angiography and Interventions; TVR, tricuspid valve repair.

Patients who died within 30 days of ICU admission (*Table 1*) had higher rates of sepsis (23.3 vs. 5.2%, *P* < 0.001 in the training and 25.0 vs. 5.6%, *P* < 0.001 in the validation cohort), were more frequently resuscitated prior to admission (53.3 vs. 12.6%, *P* < 0.001 in the training and 36.0 vs. 6.0%, *P* < 0.001 in the validation cohort), and showed higher rates of intubation at admission (60.0 vs. 18.8%, *P* < 0.001 in the training cohort and 71.0 vs. 15.1%, *P* < 0.001 in the validation cohort), mechanical circulatory support (16.7 vs. 5.5%, *P* = 0.048 in the training and 29.0 vs. 3.7%, *P* < 0.001 in the validation cohort), treatment with vasopressors (83.3 vs. 29.5%, *P* < 0.001 in the training and 85.0 vs. 29.6%, *P* < 0.001 in the validation cohort), renal insufficiency (serum creatinine 1.7 ± 1.6 vs. 1.1 ± 0.7 mg/dL, *P* < 0.001 in the training and 1.7 ± 1.3 vs. 1.1 ± 0.6 mg/dL, *P* < 0.001 in the validation cohort), and SCAI stages ≥ C (*P* < 0.001 for both cohorts). Additionally, non-survivors in the training cohort showed lower rates of ACS (30.0 vs. 50.8%, *P* = 0.044).

**Figure 2:**
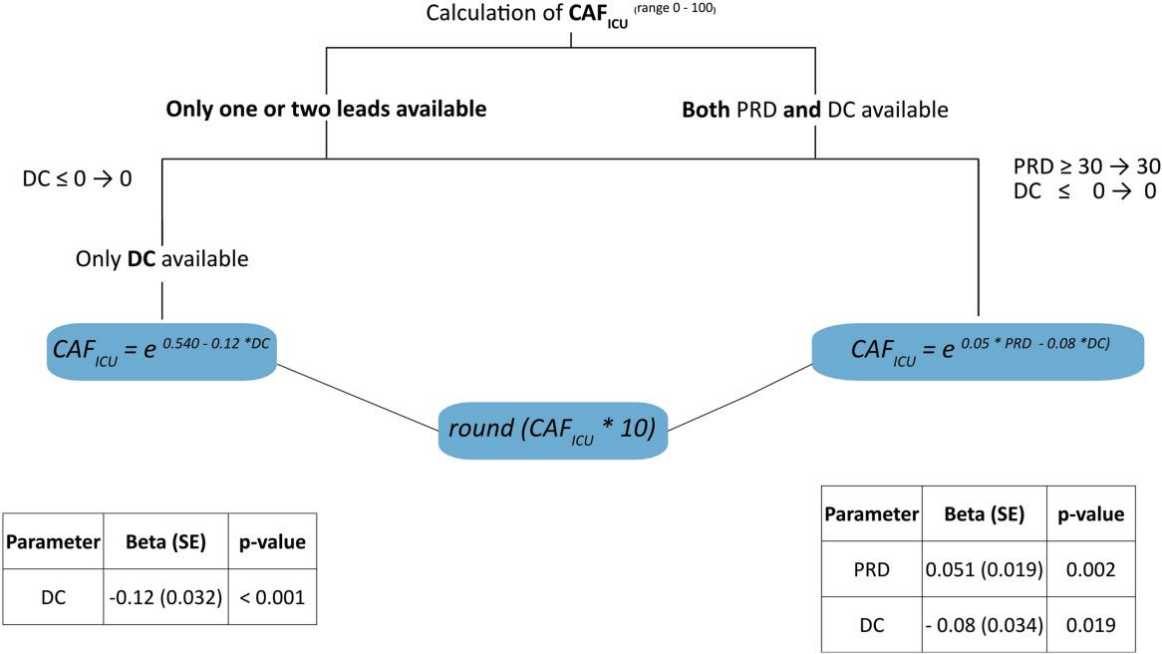
Calculation of cardiac autonomic function score (CAF_ICU_) in the training cohort, based on the formula = round [10 × exp(beta_PRD_ × PRD + beta_DC_ × DC)]); if PRD is not available, CAF_ICU_ is calculated according to the formula CAF_ICU_ = round [10 × (*C* × beta_DC_ + beta_DC_ × DC)], where *C* = 4.5. DC, deceleration capacity of heart rate; PRD, periodic repolarization dynamics.

**Figure 3:**
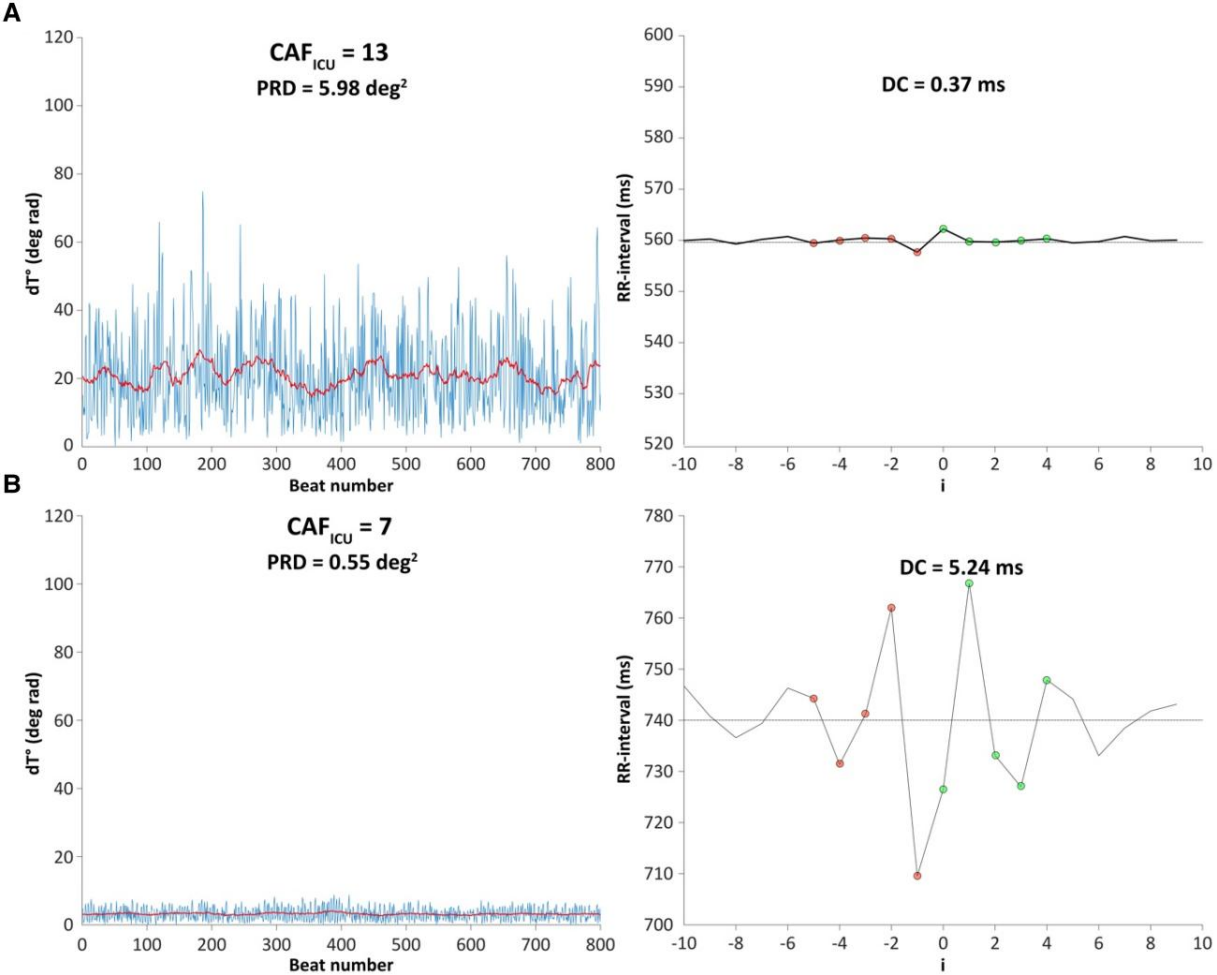
Typical biosignals of periodic repolarization dynamics and deceleration capacity of heart rate and their corresponding cardiac autonomic function scores in a patient dying 1 day after intensive care unit admission (*A*) and a patient surviving the follow-up period (*B*).

**Figure 4:**
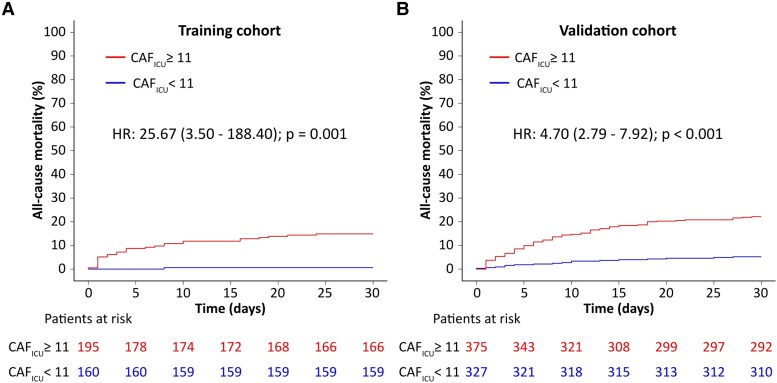
Cumulative 30-day mortality rates in the training (*A*) and validation (*B*) cohorts stratified by cardiac autonomic function score (CAF_ICU_) ≥ 11 units and <11 units.

Among the parameters of HRV (PRD is not considered an HRV parameter), DC was the only significant predictor of mortality in the training cohort (see Supplementary material online, *Table S2*). Deceleration capacity of heart rate was significantly lower (1.6 ± 3.2 vs. 4.1 ± 5.3 ms, *P* < 0.001 in the training cohort and 1.5 ± 2.3 vs. 4.2 ± 4.6 ms, *P* < 0.001 in the validation cohort) and PRD significantly higher (12.9 ± 7.0 vs. 7.2 ± 8.7 deg^2^, *P* = 0.016 in the training and 9.3 ± 8.8 vs.7.8 ± 8.1 deg^2^, *P* = 0.031 in the validation cohort, *Table 1*) in non-survivors. Multivariable Cox regression analysis in the training cohort was used to calculate the beta coefficients for PRD and DC, which were subsequently used for the calculation of CAF_ICU_ (*Figure 2*). CAF_ICU_ was significantly higher (14 ± 8 vs. 11 ± 6 units, *P* < 0.001 in the training and 13 ± 6 vs. 10 ± 6 units, *P* < 0.001 in the validation cohort, *Table 1*) in non-survivors. *Figure 3* illustrates typical PRD and DC signals and their corresponding CAF_ICU_ values in a patient dying 1 day after ICU admission (*Figure 3A*) and a patient surviving the 30-day follow-up period (*Figure 3B*). The distribution of CAF_ICU_ in the training cohort is illustrated in Supplementary material online, *Figure S1*. CAF_ICU_ was dichotomized at the median value of 11 units. In the training cohort, patients with CAF_ICU_ ≥ 11 units had a 30-day mortality rate of 14.9% (95% CI 9.7–19.7%; 29 deaths) compared with a 30-day mortality rate of 0.6% (95% CI 0–1.8%; 1 death) for patients with CAF_ICU_ < 11 units (HR 25.67; 95% CI 3.50–188.40%; *P* < 0.001; *Figure 4A*). In the validation cohort, there was no significant difference in the level of CAF_ICU_ (11 ± 5 units) compared with the training cohort (11 ± 7 units, *P* = 0.642). In the validation cohort, 375 (53.4%) patients showed CAF_ICU_ values ≥ 11 units. The 30-day mortality rates among patients with CAF_ICU_ ≥ 11 vs. < 11 units were 22.1% (95% CI 17.8–26.2%; 83 deaths) vs. 5.2% (95% CI 2.8–7.6%; 17 deaths), respectively (HR 4.70; 95% CI 2.79–7.92%; *P* < 0.001; *Figure 4B*). There was no interaction between the performance of CAF_ICU_ and the type of cohort (training vs. validation; *P*-value for interaction = 0.102). The area under the ROC curve for CAF_ICU_ was 0.752 in the training and 0.697 in the validation cohort with a Youden’s *J* statistic of 0.456 and 0.345, respectively (see Supplementary material online, *Figure S2*). The calibration plots for the training and validation cohorts are illustrated in Supplementary material online, *Figure S3*.

**Figure 5:**
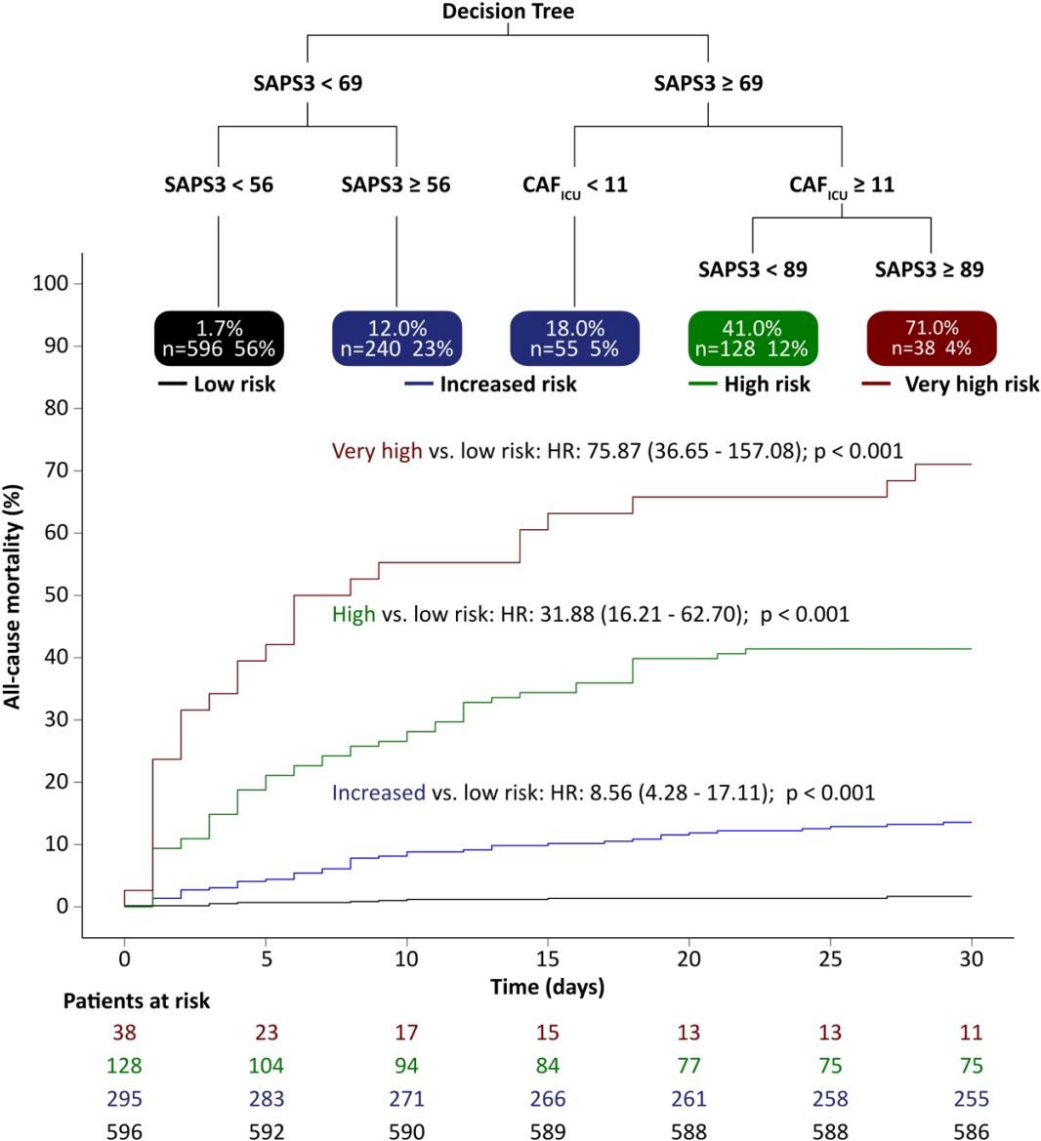
Decision-tree based classification of patients into risk groups based on the Simplified Acute Physiology Score 3 (SAPS3) and cardiac autonomic function score (CAF_ICU_) scores. Low risk: SAPS3 < 56; increased risk: SAPS3 ≥ 56 and <69 or SAPS3 ≥ 69 and CAF_ICU_ < 11; high risk: SAPS3 ≥ 69 and <89 and CAF_ICU_ ≥ 11; and very high risk: SAPS3 ≥ 89 and CAF_ICU_ ≥ 11.

In the pooled cohort, CAF_ICU_ ≥ 11 units was a strong and independent predictor of all-cause mortality. CAF_ICU_ ≥ 11 units also predicted the composite endpoint of mortality, VF, and VT (*Table 2*). The inclusion of CAF_ICU_ in a Cox regression model, which also included SAPS3, significantly improved the classification between survivors and non-survivors (increase in IDI 0.033; 95% CI 0.010–0.061; *P* < 0.001, increase in continuous NRI 0.360; 95% CI 0.282–0.431; *P* < 0.001, as well as a median improvement of 0.060; 95% 0.022–0.090; *P* < 0.001). These results were consistent in both the pooled and the validation cohorts (*Table 2* and Supplementary material online, *Table S3*). *Figure 5* illustrates decision-tree based classification of patients into risk groups based on SAPS3 and CAF_ICU_ sores. According to this analysis four risk groups could be defined (very low risk: SAPS3 < 56; low risk SAPS3 ≥ 56 and <69 or SAPS3 ≥ 69 and CAF_ICU_ < 11; intermediate risk: SAPS3 ≥ 69 and < 89 and CAF_ICU_ ≥ 11; and high risk: SAPS3 ≥ 89 and CAF_ICU_ ≥ 11). Every step-up increase in the risk score was associated with a 3.8-fold increased risk for death within 30 days after ICU admission (HR per point increase: 3.81; 95% CI 3.21–4.52; *P* < 0.001). The cumulative risk for increased, high, and very high-risk groups compared with the low-risk group is illustrated in *Figure 5*.

**Table 2:** Univariable and multivariable Cox regression analysis for prediction of 30-day all-cause mortality (A) and the composite of 30-day all-cause mortality, ventricular fibrillation, and ventricular tachycardia (B) in the pooled cohort (*N* = 1057) cohort

	Univariable analysis		Multivariable analysis	
	Hazard ratio (95% CI)	*P*-value	Hazard ratio (95% CI)	*P*-value
**A. 30-day all-cause mortality**				
SAPS3 per unit increase	1.07 (1.06–1.09)	< 0.001	1.07 (1.06–1.08)	<0.001
CAF_ICU_ ≥ 11 units	5.83 (3.54–9.59)	< 0.001	3.25 (1.96–5.40)	<0.001
			**Change (95% CI)**	***P*-value**
IDI			0.033 (0.010–0.061)	<0.001
Continuous NRI			0.360 (0.282–0.431)	<0.001
Median improvement			0.060 (0.022–0.090)	<0.001
**B. 30-day all-cause mortality, VF, and VT**				
SAPS3 per unit increase	1.05 (1.04–1.06)	< 0.001	1.05 (1.04–1.06)	<0.001
CAF_ICU_ ≥ 11 units	3.07 (2.17–4.33)	< 0.001	2.04 (1.43–2.91)	<0.001
			**Change (95% CI)**	***P*-value**
IDI			0.022 (0.008–0.039)	<0.001
Continuous NRI			0.272 (0.200–0.338)	<0.001
Median improvement			0.053 (0.011–0.082)	<0.001

CAF_ICU_, cardiac autonomic function score in the ICU; IDI, integrated discrimination index; NRI, net reclassification index; SAPS3, Simplified Acute Physiology Score 3.

**Figure 6:**
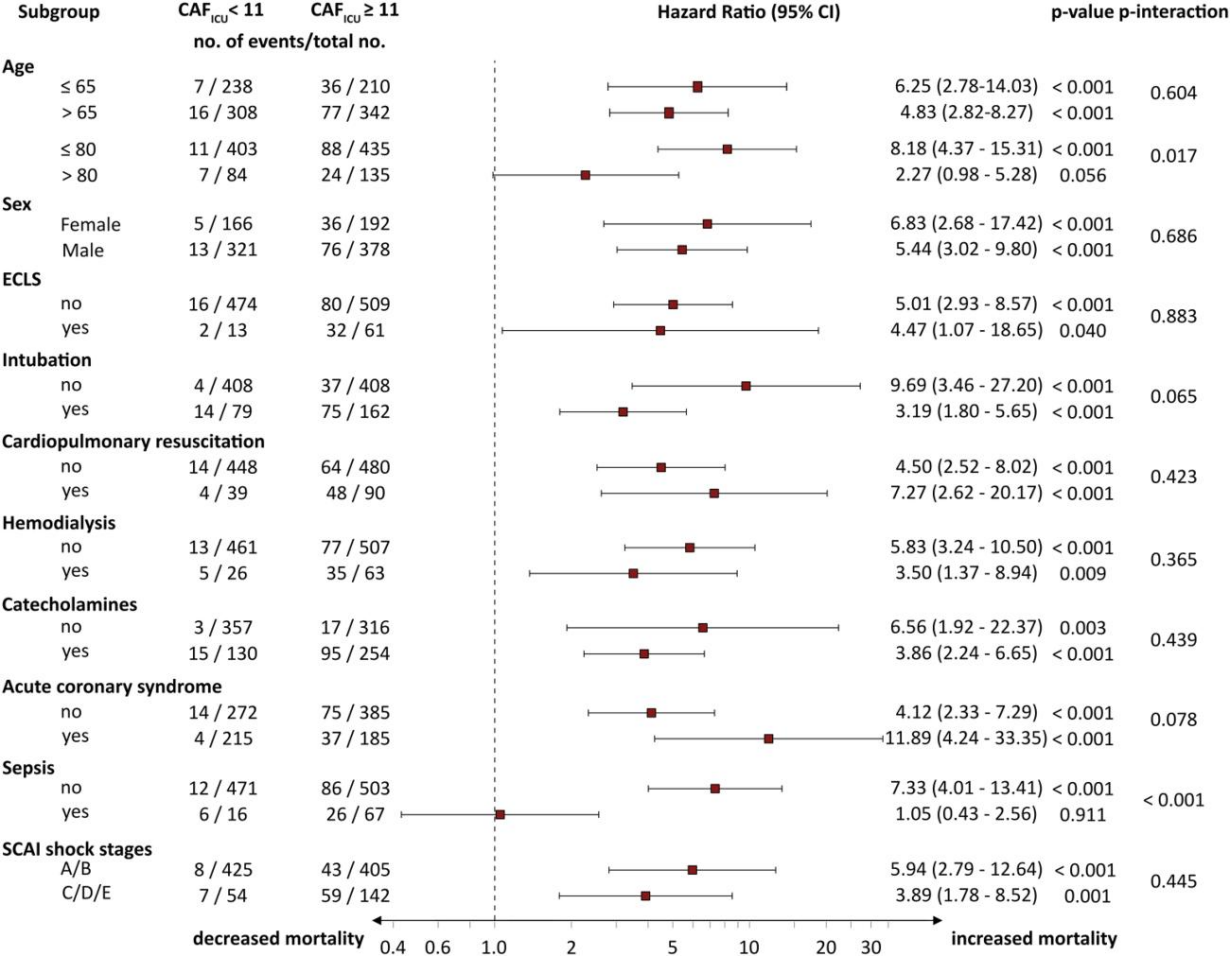
Subgroup analysis stratified by cardiac autonomic function score (CAF_ICU_) ≥ 11 units in the pooled cohort. ECLS, extracorporeal life support.

Subgroup analysis in the pooled cohort revealed that the predictive value of CAF_ICU_ was consistent among different subgroups (*Figure 6*). The only significant interactions were identified in patients with and without sepsis (*P* for interaction < 0.001) and patients aged ≥/<80 years. There was a trend for better performance of CAF_ICU_ among non-intubated patients (*P* for interaction = 0.065) and patients with ACS (*P* for interaction = 0.078). The subgroup analysis of the validation cohort was consistent with the findings from the pooled cohort (see Supplementary material online, *Figure S4*). It is worth noting that CAF_ICU_ was a significant predictor of mortality in patients treated with ECLS (see Supplementary material online, *Figure S5*), as well as across all SCAI shock stages (*P* for interaction 0.445, Supplementary material online, *Tables S4*–*S6*).

In both cohorts (see Supplementary material online, *Table S7*), patients excluded from the study were older (77 ± 15 vs. 67 ± 24 years, *P* < 0.001 in the training and 77 ± 15 vs. 70 ± 22 years, *P* < 0.001 in the validation cohort), suffered less frequently from ACS (20.0 vs. 49.0% in the training and 17.6 vs. 32.2%, *P* < 0.001 in the validation cohort), more frequently underwent transfemoral mitral or tricuspid valve repair (15.4 vs. 3.1%, *P* < 0.001 in the training and 11.5 vs. 3.7%, *P* < 0.001 in the validation cohort), were more frequently hospitalized due to acute decompensated heart failure (22.9 vs. 12.7% in the training, *P* = 0.002 and 25.8 vs. 15.4%, *P* < 0.001 in the validation cohort), and also showed higher creatinine values (1.4 ± 0.9 vs. 1.2 ± 0.7 mg/dL, *P* = 0.001 in the training and 1.3 ± 1.0 vs. 1.1 ± 0.7 mg/dL, *P* < 0.001 in the validation cohort). Excluded patients more frequently underwent aortic valve replacement (23.4 vs. 18.4%, *P* = 0.044) in the validation cohort. Sensitivity analysis using propensity score–derived weighted Cox regression analysis showed that the results remained consistent after matching the included with the excluded patients (see Supplementary material online, *Table S8*). Supplementary material online, *Figure S6* illustrates the interaction between inclusion month and mortality (i), resuscitation (ii), sepsis (iii), and intubation (iv). Among the 701 excluded patients, 214 patients presented with AF. The baseline characteristics of these patients compared with patients presenting in sinus rhythm are depicted in Supplementary material online, *Table S9*. Heart rate variability and CAF_ICU_ cannot be quantified in patients presenting with AF. Periodic repolarization dynamics was significantly higher in patients with AF than SR (14.14; IQR 8.54 vs. 7.88; IQR 8.45 deg^2^, *P* < 0.001). In the subgroup of AF patients, PRD was not statistically predictive of mortality (see Supplementary material online, *Table S10*). The interaction between PRD and presence of AF for prediction of all-cause mortality was not statistically significant.

## Discussion

The findings of our study indicate that automated ECG-based cardiac autonomic function testing by means of PRD and DC using routine monitor data is feasible and is a strong and independent predictor of short-term mortality in the cardiac ICU. Moreover, this method can be effectively combined with established ICU risk scores, such as SAPS3 to identify low, increased, high, and very high-risk patients. This dynamic, multilevel risk stratification strategy is of great clinical importance for various reasons. First, the correct selection of very low-risk patients, who can be transferred to general wards could lead to ICU decongestion, as well as improve patient outcomes by reducing adverse effects associated with ICU treatment such as multi-resistant bacterial infections or post-intensive care syndrome.^22,23^ Moreover, identification of patients with a futile prognosis might help redirecting invasive therapies to patients ‘who can still be saved’. A case in point is the recent ECLS-SHOCK^24^ trial, which demonstrated that treatment with ECLS in infarct-related cardiogenic shock failed to reduce mortality. The ECLS-SHOCK trial underscores the need to properly identify patients with non-favourable, but reversible outcome, in whom aggressive invasive therapies are still reasonable. Our method was predictive of 30-day mortality in patients treated with ECLS and could prove a useful tool for risk stratification in patients with cardiogenic shock.

Several scoring systems have been developed over time for risk stratification of patients in the ICU.^1–4^ In this study, we decided to use SAPS3, as an established ICU score for several reasons. First, a head-to-head comparison between SOFA, APACHE II, and SAPS3 showed that SAPS3 was superior for prediction of short-term mortality in the surgical ICU.^25^ Second, at the time of study conception, SAPS3 was the only risk score tested in the cardiac ICU.^26^ Third, SAPS3 includes the highest heart rate within 1 h after admission as co-variate for prediction of mortality. Although SAPS3 shows a very strong association with mortality, there are no established cut-off values to guide triaging of invasive therapies. Moreover, SAPS3 does not consider the autonomic tone of the patient. Here, we propose a risk stratification algorithm based on different cut-off values of SAPS3 and CAF_ICU_ for risk stratification of patients in the cardiac ICU.

In this study, we quantified the cardiac autonomic tone of the patient using a combination of DC and PRD. This method has several advantages compared with other methods based on HRV. First, among other HRV parameters, DC was the only predictor of mortality in multivariable analysis. Second, combining DC and PRD provides a global evaluation of cardiac autonomic function, by assessment of both vagal (DC) and sympathetic (PRD) function. Both DC and PRD have been validated for short ECG recordings of 20 min or more, which makes them better suited for continuous monitoring and dynamic risk stratification in ICUs. As discussed in an editorial about challenges of HRV research in the ICU, other widely used parameters like the SDNN can only be validly calculated over 24 h.^27^ In this study, we assessed CAF_ICU_ at night (between 2:00 and 2:30 a.m.) to achieve standardized conditions. However, there is no limitation for assessment of CAF_ICU_ at different times and for different intervals. Thus, CAF_ICU_ could provide a continuous, dynamic risk assessment tool in the cardiac ICU. Another strength of our methodology is the fully automated approach, using ECG signals acquired from routine monitoring. The algorithm used to calculate CAF_ICU_ could directly be implemented into monitoring systems allowing autonomic risk stratification in everyday clinical practice.

It cannot be neglected that calculation of HRV requires an intact function of the sinus node and therefore patients presenting with AF cannot be evaluated by means of CAF_ICU_. Little evidence is present with respect to the prognostic value of PRD in patients with AF. DANISH was the only trial, where evaluation of PRD did not exclude patients presenting with AF.^6^ In the PRD substudy of DANISH, 162 out of 748 patients (22%) were in AF during the Holter ECG. In both DANISH and eICU, PRD was significantly higher in patients with AF than patients in sinus rhythm. In both studies, no significant interaction was found between AF and PRD for the prediction of all-cause mortality. However, in both studies, the prognostic value of PRD for predicting all-cause mortality in patients with AF did not reach the level of statistical significance. Possible explanations for this finding might be the limited statistical power for this subgroup analysis, the older age of patients presenting with AF, as well as a presumable lower predictive power of PRD in this subgroup.

The general belief is that autonomic risk stratification is a useful tool for prediction of SCD. However, SCD is not a major issue in ICUs, as the patients are continuously monitored, and ventricular arrhythmias can be promptly and successfully treated. Cardiac death in ICUs is usually related to end-stage heart failure. In previous studies, we could show that PRD was a strong predictor not only of SCD, but also of acute decompensated heart failure and cardiac non-SCD.^6,7,28^ Moreover, PRD could identify patients in whom invasive therapies, like implantation of a cardioverter defibrillator, could reduce mortality.^6,7,28^ We therefore believe that assessment of CAF_ICU_ might timely identify patients at high risk for cardiogenic shock, who may benefit from preemptive medical or interventional therapies. In this study, the predictive value of PRD was present across all SCAI shock stages. As we recently learned from the ECLS-SHOCK trial, a part of the benefit accomplished by ECLS implantation was counterbalanced by an increased rate of bleeding complications,^24^ which contributed to the neutral results of the study with respect to reduction of mortality. Prompt identification of ECLS candidates and ‘elective’ ECLS implantation might lead to a significant reduction of bleeding complications and therefore could finally make ECLS therapy beneficial.

The all-comers collective from our ICU is heterogeneous concerning the severity of the patients’ conditions ranging from patients who are awake and stable without vasoactive medication, i.e. after ST-elevation myocardial infarction to patients in severe cardiogenic shock requiring ECLS and high levels of catecholamines, as well as patients after CPR. Subgroup analysis revealed that CAF_ICU_ could successfully identify high-risk patients in all subgroups, except for patients older than 80 years and patients with sepsis. Septic patients showed higher levels of CAF_ICU_, compared with non-septic patients (13 ± 6.5 vs. 11 ± 6; *P* < 0.001; 81% of the patients were classified as high risk based on the CAF_ICU_ cut-off value of ≥ 11 units). While a generalized activation of the sympathetic nervous system as well as vagal disbalance are both expected in septic patients, it appears that this was not predictive of mortality. This finding is reasonable, as it is known that other co-variates (for instance, patient comorbidities, inflammatory lab values) are more important prognostic markers in sepsis. Another explanation might be that a higher cut-off value or adjusted algorithm may be necessary in septic shock, because septic patients show a generalized sympathetic activation. These findings should be further evaluated in larger general ICU cohorts. It is worth noting that there was no significant interaction between the predictive value of CAF_ICU_ and the treatment with catecholamines. A possible explanation might be that CAF_ICU_ most probably detects the endogenous level of sympathetic activation.

Our study has several limitations. First, data were retrospectively collected. Second, patients not in sinus rhythm were excluded from the study. Adjudication of baseline characteristics using a propensity score analysis confirmed our initial results. However, our results cannot be extrapolated to patients presenting with AF. Heart rate variability calculation requires an intact sinus node. Moreover, there is limited evidence regarding the predictive value of PRD in patients presenting with AF. Third, there was a difference in mortality rates between training and validation cohorts (8.5 vs. 14.2%). This difference can be most probably attributed to seasonal variation in the incidence of sepsis, CPR, as well as the rate of ACS^29^ and a progressive transformation of the disease spectrum treated at our institution towards interventional valve procedures. Moreover, this finding might correlate with the progressive transformation of the treated population at our institution towards reduced ACS treatment and increased valve procedures. Fourth, at the time of study conception, SAPS3 was the only risk score tested in the cardiac ICU.^26^ In recent years, other scores, including SOFA,^30^ APACHE III, APACHE IV, and Mayo cardiac ICU admission scores (M-CARS), have been validated in unselected patients in the cardiac ICU.^31^ The incremental predictive value of CAF_ICU_ over additional ICU scores should be evaluated in future studies. Finally, the applicability of our findings to non-cardiac ICUs, as well as in patients with sepsis may be limited, as CAF_ICU_ was not predictive of mortality in the subgroup of septic patients.

## Conclusions

CAF_ICU_ is a strong and independent predictor of short-term mortality in patients treated in the cardiac ICU. CAF_ICU_ can be automatically calculated for patients in sinus rhythm from routine monitor ECG recordings and its prognostic value is additive to established ICU risk scores, such as SAPS3. The combination of SAPS3 with CAF_ICU_ can classify patients into very low, low, intermediate, and high-risk groups. Future studies should focus on the development of novel techniques, which will allow the calculation of CAF_ICU_ in patients with AF. Moreover, randomized trials are needed to test whether a risk stratification based on CAF_ICU_ can successfully guide medical and interventional treatment in cardiac intensive care medicine.

## Lead author biography


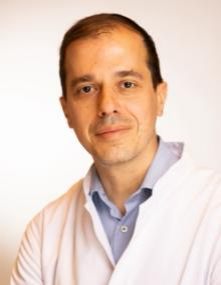


Prof. Konstantinos D. Rizas, an invasive cardiologist and electrophysiologist, is the head of the Department of Cardiology at the University Hospital of Munich (LMU)—Campus Innenstadt. His research focuses on translational digital medicine, biosignal analysis, and development of novel digital biomarkers to predict and prevent cardiovascular diseases.

## Supplementary Material

ztaf038_Supplementary_Data

## Data Availability

The source code of CAF_ICU_ and all R-scripts can be accessed upon request for invitation via https://osf.io/4q7ma/?view_only=977a7f63ad1948f4a6a74cdccb0a9a09. The data are not publicly available because they contain patient data, consented for research use by the eICU investigators. Making the data publicly available without additional consent or ethical approval might compromise patients’ privacy and the original ethical approval. If other investigators are interested in performing additional analyses, requests can be made to the corresponding author (K.D.R) and analyses will be performed in collaboration with the Ludwig-Maximilians-University Munich, Germany.
