# Supplementary material for: Cardiac autonomic function score: a novel risk stratification tool in the cardiac intensive care unit based on periodic repolarization dynamics and deceleration capacity of heart rate (LMU-eICU study)
Source: Eur Heart J Digit Health. 2025 Apr 30;6(4):822–32. doi: 10.1093/ehjdh/ztaf038 (PMC12282351; doi:10.1093/ehjdh/ztaf038)
Supplement: ztaf038_Supplementary_Data [file ztaf038_supplementary_data.docx]

**Supplementary Material**

**Cardiac Autonomic Function score: a novel risk stratification tool in the cardiac intensive care unit based on periodic repolarization dynamics and deceleration capacity of heart rate (LMU-eICU pilot study)**

Mathias Klemm^1,2^, Lukas von Stülpnagel^1,2,3^, Valentin Ostermaier^1^, Carsten Theurer^1^, Laura E Villegas Sierra^1,2^, Felix Wenner^1,2^, Elodie Eiffener^1,2^, Aresa Krasniqi^1,2^, Konstantinos Mourouzis^1,2^, Lauren E. Sams^1,2^, Luisa Freyer^1,2^, Steffen Massberg^1,2^, Axel Bauer^3^, Konstantinos D. Rizas^1,2^

^1^Medizinische Klinik und Poliklinik I, LMU Klinikum, Ludwigs-Maximilians-University Munich, Germany;

^2^German Centre for Cardiovascular Research (DZHK), Germany

^3^University Hospital for Internal Medicine III, Medical University of Innsbruck, Austria

**Address for correspondence**

Prof. Dr. med. Konstantinos Rizas

Medizinische Klinik und Poliklinik I,

University Hospital Munich,

Ludwig-Maximilians University Munich

Ziemsenstr.5, 80336 Munich, Germany

Email: Konstantinos.Rizas@med.uni-muenchen.de

**Supplementary Table 1** Comparison of baseline characteristics between training and validation cohorts.

|  | **Training (N=355)** | **Validation (N=702)** | **p-Value** |
| --- | --- | --- | --- |
| **Age, years (IQR)** | 67 (24) | 70 (22) | 0.003 |
| **BMI, kg/m^2^ (IQR)** | 26.0 (6.0) | 26.1 (5.8) | 0.626 |
| **Female sex (%)** | 125 (35.2%) | 233 (33.2%) | 0.557 |
| **ACS (%)** | 174 (49.0%) | 226 (32.2%) | <0.001 |
| **ADHF (%)** | 45 (12.7%) | 108 (15.4%) | 0.283 |
| **AVR (%)** | 36 (10.1%) | 129 (18.4%) | <0.001 |
| **MVR/TVR (%)** | 11 (3.1%) | 26 (3.7%) | 0.743 |
| **Sepsis (%)** | 24 (6.8%) | 59 (8.4%) | 0.421 |
| **Circulatory support (%)** | 123 (34.6%) | 265 (37.7%) | 0.314 |
| **Catecholamines (%)** | 121 (34.1%) | 263 (37.5%) | 0.265 |
| **ECLS (%)** | 23 (6.5%) | 51 (7.3%) | 0.721 |
| **Hypertension (%)** | 238 (67.0%) | 472 (67.2%) | 0.758 |
| **CAD (%)** | 256 (72.1%) | 461 (65.7%) | 0.039 |
| **CPR prior to admission (%)** | 57 (16.1%) | 72 (10.3%) | 0.009 |
| **Intubation (%)** | 79 (22.3%) | 162 (23.1%) | 0.805 |
| **Diabetes (%)** | 85 (23.9%) | 201 (28.6%) | 0.159 |
| **Smokers (%)** | 156 (43.9%) | 254 (36.2%) | 0.002 |
| **COPD (%)** | 40 (11.3%) | 62 (8.8%) | 0.218 |
| **Dialysis (%)** | 11 (3.1%) | 78 (11.1%) | <0.001 |
| **Max Creatinine within 24h, mg/dl** | 1.2 (0.7) | 1.1 (0.7) | 0.114 |
| **SAPSIII Score (IQR)** | 52.0 (24.0) | 53.0 (19.0) | 0.602 |
| **Autonomic risk score (IQR)** | 11 (7) | 11 (5) | 0.642 |

ACS, acute coronary syndrome; ADHF, acute decompensated heart failure; AVR, aortic valve replacement; BMI, body mass index; CAD, coronary artery disease; CAF_ICU_, cardiac autonomic function score in the ICU; CPR, cardiopulmonary resuscitation; DC, deceleration capacity of heart rate; ECLS, Extracorporeal circulatory life support; MVR, mitral valve repair; PRD, periodic repolarization dynamics; SAPS3, Simplified Acute Physiology Score 3; TVR, tricuspid valve repair

**Supplementary Table 2** Univariable and multivariable analysis for prediction of mortality in the training cohort based on parameters of heart rate variability. All parameters have been standardized according to their mean values and standard deviation. Parameters with p < 0.10 in univariable analysis have been included in the multivariable model.

|  | **Univariable analysis** | | **Multivariable analysis** | |
| --- | --- | --- | --- | --- |
|  | **Hazard Ratio (95% CI)** | **p-value** | **Hazard Ratio (95% CI)** | **p-value** |
| **Mean HR per SD increase** | 1.64 (1.17 – 2.28) | 0.004 | 1.06 (0.72 – 1.57) | 0.756 |
| **SDNN per SD increase** | 0.69 (0.43 – 1.11) | 0.124 |  |  |
| **HRVI per SD increase** | 0.31 (0.16 – 0.62) | < 0.001 | 0.65 (0.25 – 1.73) | 0.389 |
| **RMSSD per SD increase** | 0.80 (0.52 – 1.22) | 0.301 |  |  |
| **VLF per SD increase** | 0.40 (0.15 – 1.07) | 0.068 | 1.08 (0.61 – 1.90) | 0.789 |
| **LF per SD increase** | 0.84 (0.47 – 1.50) | 0.562 |  |  |
| **HF per SD increase** | 1.01 (0.70 – 1.46) | 0.962 |  |  |
| **LF/HF per SD increase** | 0.89 (0.58 – 1.36) | 0.590 |  |  |
| **DC per SD increase** | 0.66 (0.53 – 0.82) | < 0.001 | 0.42 (0.19 – 0.94) | 0.035 |

DC, deceleration capacity of heart rate; HF, high frequency band of heart rate variability; HRV, heart rate variability index; LF, low frequency band of heart rate variability RMSSD, root mean square of successive differences between normal heartbeats; SDNN, standard deviation of normal RR-intervals; VLF, very low frequency band of heart rate variability

**Supplementary Table 3** Univariable and multivariable Cox regression analysis for prediction of 30-day all-cause mortality (A) and the composite of 30-day all-cause mortality, ventricular fibrillation and ventricular tachycardia (B) in the validation cohort (N = 702)

|  | **Univariable analysis** | | **Multivariable analysis** | |
| --- | --- | --- | --- | --- |
|  | **Hazard Ratio (95% CI)** | **p-value** | **Hazard Ratio (95% CI)** | **p-value** |
|  | 1. **30-day all-cause mortality** | | | |
| **SAPS3 per unit increase** | 1.08 (1.07 – 1.09) | <0.001 | 1.07 (1.06 – 1.09) | <0.001 |
| **CAF_ICU_ ≥ 11 units** | 4.70 (2.79 – 7.92) | <0.001 | 2.28 (1.33 – 3.93) | 0.003 |
|  |  |  | **Change (95 % CI)** | **p-value** |
| **IDI** |  |  | 0.016 (0.001 – 0.044) | 0.027 |
| **Continuous NRI** |  |  | 0.335 (0.209 – 0.416) | 0.007 |
| **Median improvement** |  |  | 0.045 (0.012 – 0.079) | <0.001 |
|  | 1. **30-day all-cause mortality, VF and VT** | | | |
| **SAPS3 per unit increase** | 1.05 (1.04 – 1.06) | <0.001 | 1.05 (1.04 – 1.06) | <0.001 |
| **CAF_ICU_ ≥ 11 units** | 2.48 (1.70 – 3.62) | <0.001 | 1.58 (1.07 – 2.35) | 0.023 |
|  |  |  | **Change (95 % CI)** | **p-value** |
| **IDI** |  |  | 0.010 (0.000 – 0.027) | 0.027 |
| **Continuous NRI** |  |  | 0.226 (0.021 – 0.315) | 0.040 |
| **Median improvement** |  |  | 0.037 (0.002 – 0.076) | 0.013 |

CAF_ICU_, cardiac autonomic function score in the ICU; IDI, integrated discrimination index; NRI, net reclassification index; SAPS3, Simplified Acute Physiology Score 3

**Supplementary Table 4** SCAI Shock stages for the training and validation cohorts

|  | **Training (N=355)** | **Validation (N=702)** | **p-value** |
| --- | --- | --- | --- |
| **SCAI A** | 221 (63.3%) | 487 (69.4%) | 0.010 |
| **SCAI B** | 34 (9.6%) | 88 (12.5%) | 0.168 |
| **SCAI C** | 10 (2.8%) | 12 (1.7%) | 0.348 |
| **SCAI D** | 59 (16.6%) | 41 (5.8%) | <0.001 |
| **SCAI E** | 23 (6.5%) | 51 (7.3%) | 0.697 |

**Supplementary Table 5** Multivariable Cox regression analysis using SCAI shock stages and CAF_ICU_ score for prediction of 30-day mortality in the training and validation cohorts.

|  | **Hazard Ratio (95% CI)** | **p-value** |
| --- | --- | --- |
|  | 1. **Training** | |
| **SCAI shock stages per increase** | 1.59 (1.25 – 2.03) | <0.001 |
| **CAF_ICU_ ≥ 11 units** | 16.63 (2.24 – 123.71) | 0.006 |
|  | 1. **Validation** | |
| **SCAI shock stages per increase** | 1.72 (1.52 – 1.94) | <0.001 |
| **CAF_ICU_ ≥ 11 units** | 3.65 (2.04 – 6.54) | <0.001 |

CAF_ICU_, cardiac autonomic function score in the ICU; SCAI, society for cardiovascular angiography and interventions;

**Supplementary Table 6** Cardiac autonomic function score (CAF_ICU_) ≥ 11 units as predictor of 30-day mortality stratified by SCAI Shock stages A/B vs. C/D/E in the pooled cohort.

| **CAF_ICU_ ≥ 11** | **Hazard Ration (95% CI)** | **p-value** | **P for interaction** |
| --- | --- | --- | --- |
| **SCAI A/B** | 5.92 (2.78 – 12.59) | <0.001 | 0.445 |
| **SCAI C/D/E** | 3.93 (1.79 – 8.60) | <0.001 | 0.445 |

CAF_ICU_, cardiac autonomic function score in the ICU; SCAI, society for cardiovascular angiography and interventions;

**Supplementary Table 7** Comparison of baseline characteristics of excluded and included patients in the training and validation cohorts.

|  | **Training cohort** | | | **Validation cohort** | | |
| --- | --- | --- | --- | --- | --- | --- |
|  | **Excluded (N=240)** | **Included (N=355)** | **p-Value** | **Excluded (N=461)** | **Included (N=702)** | **p-Value** |
| **Age, years (IQR)** | 77 (15) | 67 (24) | <0.001 | 77 (15) | 70 (22) | <0.001 |
| **BMI, kg/m^2^ (IQR)** | 25.0 (5.0) | 26.0 (6.0) | 0.986 | 26.2 (5.9) | 26.1 (5.8) | 0.676 |
| **Female sex (%)** | 80 (33.3%) | 125 (35.2%) | 0.700 | 137 (29.7%) | 233 (33.2%) | 0.2172 |
| **ACS (%)** | 48 (20.0%) | 174 (49.0%) | <0.001 | 81 (17.6%) | 226 (32.2%) | <0.001 |
| **ADHF (%)** | 55 (22.9%) | 45 (12.7%) | 0.002 | 119 (25.8%) | 108 (15.4%) | <0.001 |
| **AVR (%)** | 37 (15.4%) | 36 (10.1%) | 0.072 | 108 (23.4%) | 129 (18.4%) | 0.044 |
| **MVR/TVR (%)** | 37 (15.4%) | 11 (3.1%) | <0.001 | 53 (11.5%) | 26 (3.7%) | <0.001 |
| **Sepsis (%)** | 17 (7.1%) | 24 (6.8%) | 1.000 | 41 (8.9%) | 59 (8.4%) | 0.524 |
| **Circulatory**  **support (%)** | 85 (35.4%) | 123 (34.6%) | 0.956 | 171 (37.1%) | 265 (37.7%) | 0.283 |
| **Catecholamines (%)** | 84 (35.0%) | 121 (34.1%) | 0.925 | 167 (36.2%) | 263 (37.5%) | 0.370 |
| **ECLS (%)** | 10 (4.2%) | 23 (6.5%) | 0.305 | 34 (7.4%) | 51 (7.3%) | 0.722 |
| **Hypertension (%)** | 180 (75.0%) | 238 (67.0%) | 0.035 | 304 (65.9%) | 472 (67.2%) | 0.135 |
| **CPR (%)** | 146 (60.8%) | 256 (72.1%) | 0.003 | 252 (54.7%) | 461 (65.7%) | 0.033 |
| **CPR prior to admission (%)** | 30 (12.5%) | 57 (16.1%) | 0.271 | 52 (11.3%) | 72 (10.3%) | 0.343 |
| **Intubation (%)** | 44 (18.3%) | 79 (22.3%) | 0.291 | 113 (24.5%) | 162 (23.1%) | 0.170 |
| **Diabetes (%)** | 54 (22.5%) | 85 (23.9%) | 0.734 | 123 (26.7%) | 201 (28.6%) | 0.919 |
| **Smokers (%)** | 75 (31.2%) | 156 (43.9%) | 0.001 | 135 (29.3%) | 254 (36.2%) | 0.152 |
| **COPD (%)** | 37 (15.4%) | 40 (11.3%) | 0.182 | 49 (10.6%) | 62 (8.8%) | 0.160 |
| **Dialysis (%)** | 4 (1.7%) | 11 (3.1%) | 0.398 | 51 (11.1%) | 78 (11.1%) | 0.681 |
| **Max Creatinine within 24h, mg/dl (IQR)** | 1.4 (0.9) | 1.2 (0.7) | <0.001 | 1.3 (1.0) | 1.1 (0.7) | <0.001 |

ACS, acute coronary syndrome; ADHF, acute decompensated heart failure; AVR, aortic valve replacement; BMI, body mass index; CAD, coronary artery disease; CAF_ICU_, cardiac autonomic function score in the ICU; CPR, cardiopulmonary resuscitation; DC, deceleration capacity of heart rate; ECLS, Extracorporeal circulatory life support; MVR, mitral valve repair; PRD, periodic repolarization dynamics; SAPS3, Simplified Acute Physiology Score 3; TVR, tricuspid valve repair

**Supplementary Table 8** Univariable and multivariable Cox regression analysis for prediction of 30-days all-cause mortality using propensity score derived weighted Cox regression analysis.

|  | **Univariable analysis** | | **Multivariable analysis** | |
| --- | --- | --- | --- | --- |
|  | **Hazard Ratio (95% CI)** | **p-value** | **Hazard Ratio (95% CI)** | **p-value** |
|  | 1. **30-days all-cause mortality** | | | |
| **SAPS3 per unit increase** | 1.07 (1.05 – 1.08) | < 0.001 | 1.07 (1.05 – 1.08) | < 0.001 |
| **CAF_ICU_ ≥ 11 units** | 4.62 (2.42 – 8.84) | < 0.001 | 3.38 (1.65 – 6.90) | < 0.001 |
|  |  |  | **Change (95 % CI)** | **p-value** |
| **IDI** |  |  | 0.035 (0.020 – 0.053) | < 0.001 |
| **Continuous NRI** |  |  | 0.367 (0.291 – 0.423) | < 0.001 |
| **Median improvement** |  |  | 0.056 (0.030 – 0.077) | < 0.001 |

CAF_ICU_, cardiac autonomic function score in the ICU; IDI, integrated discrimination index; NRI, net reclassification index; SAPS3, Simplified Acute Physiology Score 3; Both univariable and multivariable analyses are adjusted for the propensity score

**Supplementary Table 9** Baseline characteristics of the cohort of patients in atrial fibrillation

|  | **Total (N=1271)** | **AF (N=214)** | **SR (N=1057)** | **p-value** |
| --- | --- | --- | --- | --- |
| **Age, years (IQR)** | 72 (59-80) | 79 (73-83) | 70 (56-79) | <0.001 |
| **BMI, kg/m2 (IQR)** | 26 (23-29) | 26 (23-29) | 26 (23-29) | 0.741 |
| **Male sex (%)** | 847 (66.6%) | 148 (69.2%) | 699 (66.1%) | 0.437 |
| **Acute coronary syndrome (%)** | 435 (34.5%) | 35 (16.9%) | 400 (38.0%) | <0.001 |
| **Decompensated heart failure (%)** | 217 (17.2%) | 64 (30.8%) | 153 (14.5%) | <0.001 |
| **Aortic valve replacement (%)** | 217 (17.1%) | 52 (24.3%) | 165 (15.6%) | 0.003 |
| **Mitral/Tricuspid valve repair (%)** | 90 (7.1%) | 53 (24.8%) | 37 (3.5%) | <0.001 |
| **Sepsis (%)** | 95 (7.5%) | 12 (5.6%) | 83 (7.9%) | 0.319 |
| **Catecholamines (%)** | 447 (35.2%) | 63 (29.4%) | 384 (36.3%) | 0.065 |
| **ECLS (%)** | 81 (6.4%) | 7 (3.3%) | 74 (7.0%) | 0.060 |
| **Hypertension (%)** | 874 (69.6%) | 164 (78.5%) | 710 (67.8%) | 0.003 |
| **Coronary heart disease (%)** | 196 (15.6%) | 30 (14.4%) | 166 (15.8%) | 0.223 |
| **CPR prior to admission (%)** | 144 (11.3%) | 15 (7.0%) | 129 (12.2%) | 0.039 |
| **Intubation (%)** | 273 (21.5%) | 32 (15.0%) | 241 (22.8%) | 0.014 |
| **Diabetes (%)** | 336 (26.7%) | 50 (24.0%) | 286 (27.2%) | 0.386 |
| **Smokers (%)** | 471 (38.0%) | 61 (29.9%) | 410 (39.6%) | 0.011 |
| **COPD (%)** | 131 (10.4%) | 29 (14.0%) | 102 (9.7%) | 0.086 |
| **Dialysis (%)** | 30 (2.4%) | 4 (1.9%) | 26 (2.5%) | 0.736 |
| **Max Creatinine within 24h, mg/dl (IQR)** | 1.2 (0.9-1.7) | 1.4 (1.1-2.2) | 1.1 (0.9-1.6) | <0.001 |
| **Periodic repolarization dynamics, deg^2^** | 9.09 (5.05-14.20) | 14.14 (9.99-18.53) | 7.88 (4.52-12.97) | <0.001 |
| **SCAI A** | 869 (68.4%) | 161 (75.2%) | 708 (67.0%) | 0.011 |
| **SCAI B** | 141 (11.1%) | 19 (8.9%) | 122 (11.5%) | 0.328 |
| **SCAI C** | 28 (2.2%) | 6 (2.8%) | 22 (2.1%) | 0.675 |
| **SCAI D** | 113 (8.9%) | 13 (6.1%) | 100 (9.5%) | 0.154 |
| **SCAI E** | 81 (6.4%) | 7 (3.3%) | 74 (7.0%) | 0.063 |
| **Deaths** | 154 (12.1%) | 24 (11.2%) | 130 (12.3%) | 0.743 |

AF, atrial fibrillation; BMI, body mass index; COPD, chronic obstructive pulmonary disease; CPR, cardiopulmonary resuscitation; ECLS, Extracorporeal circulatory life support; PRD, periodic repolarization dynamics; SCAI, society for cardiovascular angiography and interventions; SR, sinus rhythm

**Supplementary Table 10** Predictive value of periodic repolarization dynamics for prediction of all-cause mortality in patients with atrial fibrillation and Sinus rhythm.

|  | **Hazard Ratio (95% CI)** | **p-value** | **p-value for the interaction** |
| --- | --- | --- | --- |
| **Sinus rhythm** | 1.04 (1.02 – 1.06) | <0.001 | 0.510 |
| **Atrial fibrillation** | 1.02 (0.96 – 1.08) | 0.489 |  |

**
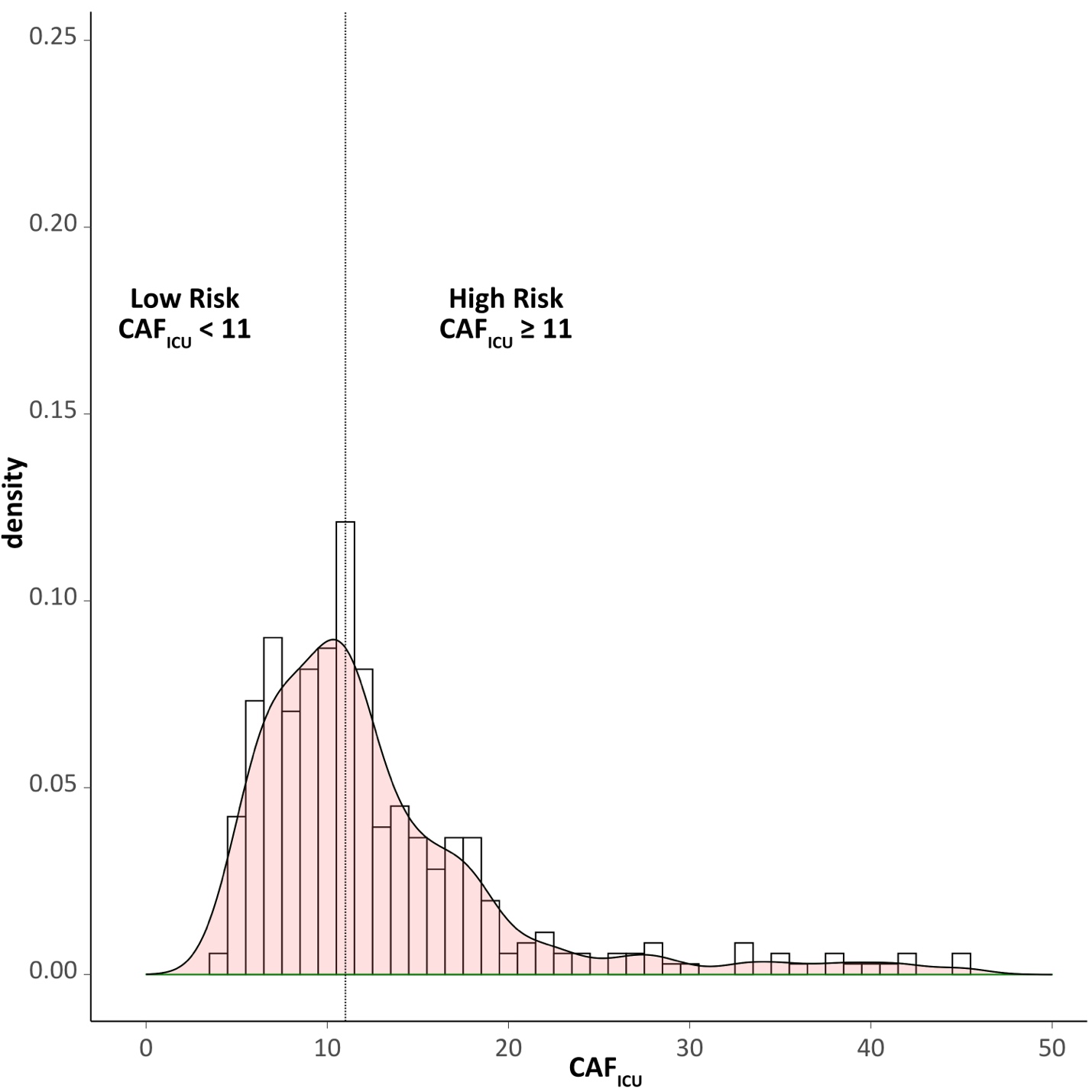
**

**Supplementary Figure 1** Distribution of the cardiac autonomic function score (CAF_ICU_) in the training cohort


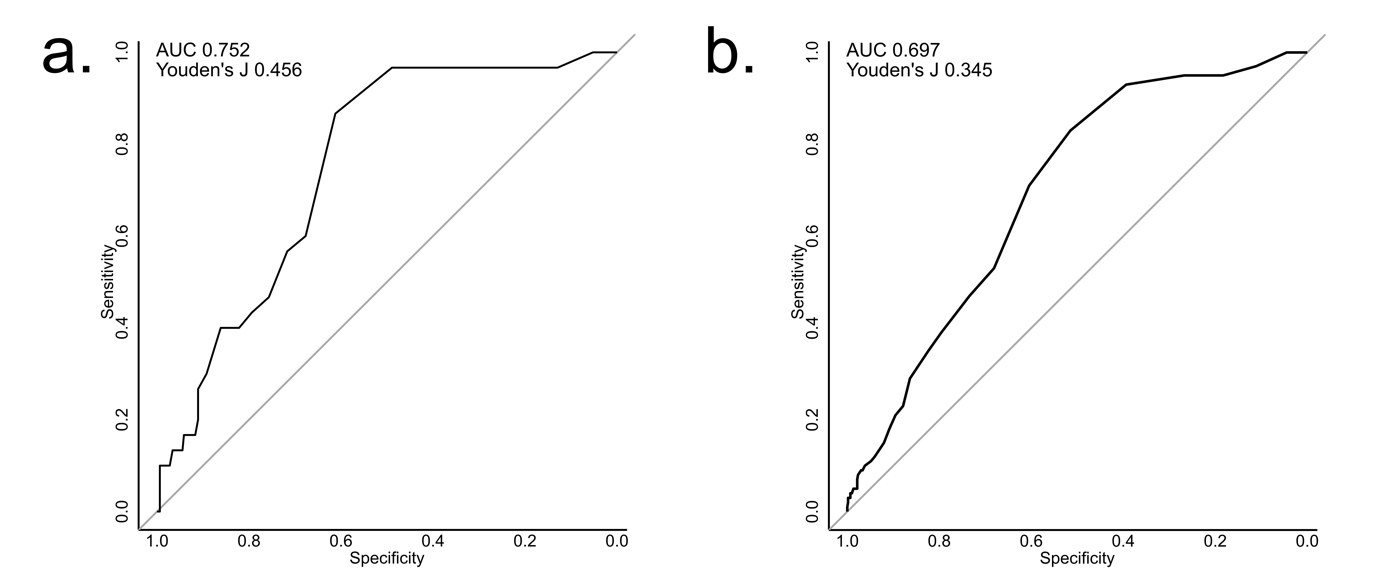


**Supplementary Figure 2:** ROC analysis for the training (A) and validation (B) cohorts for CAF_ICU_ and 30-day mortality. Youden’s J statistic is presented for the CAF_ICU_ cut off value of 11. The ideal cut off according to Youden’s statistic is 11.5 in the training and 10.5 in the validation cohort.


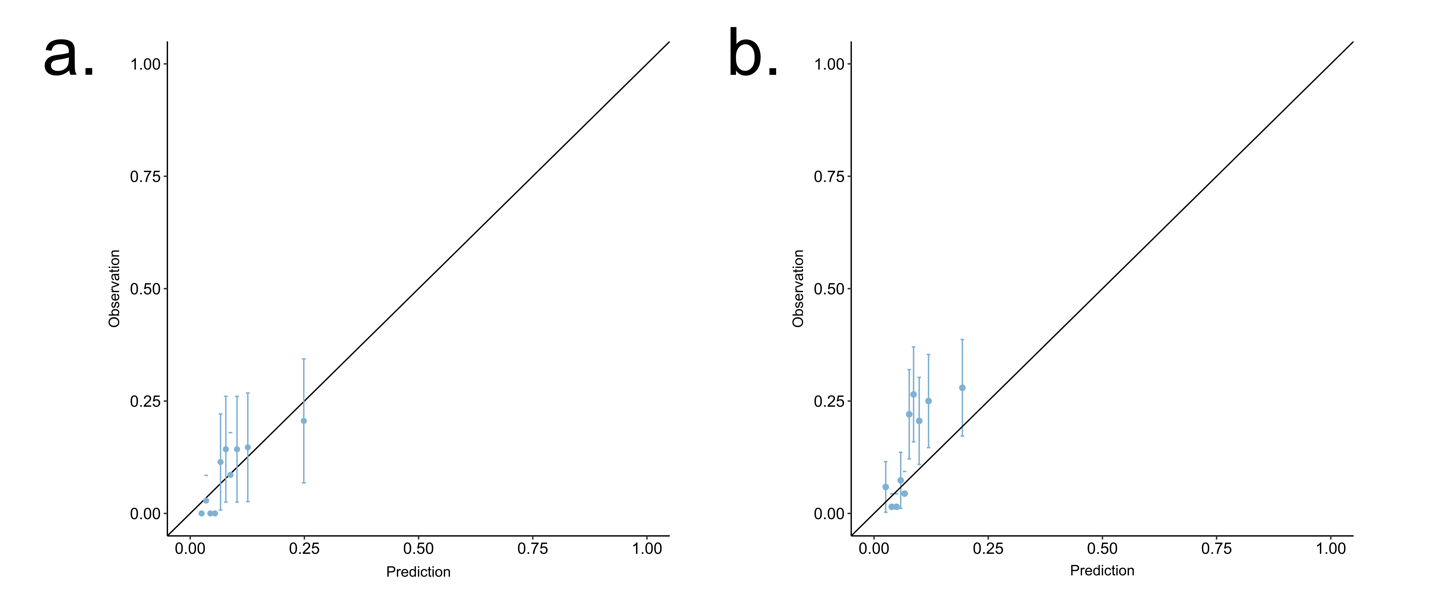


**Supplementary Figure 3:** Calibration plots for the training (a) and validation (b) cohorts for DC and PRD and 30-day mortality.

**
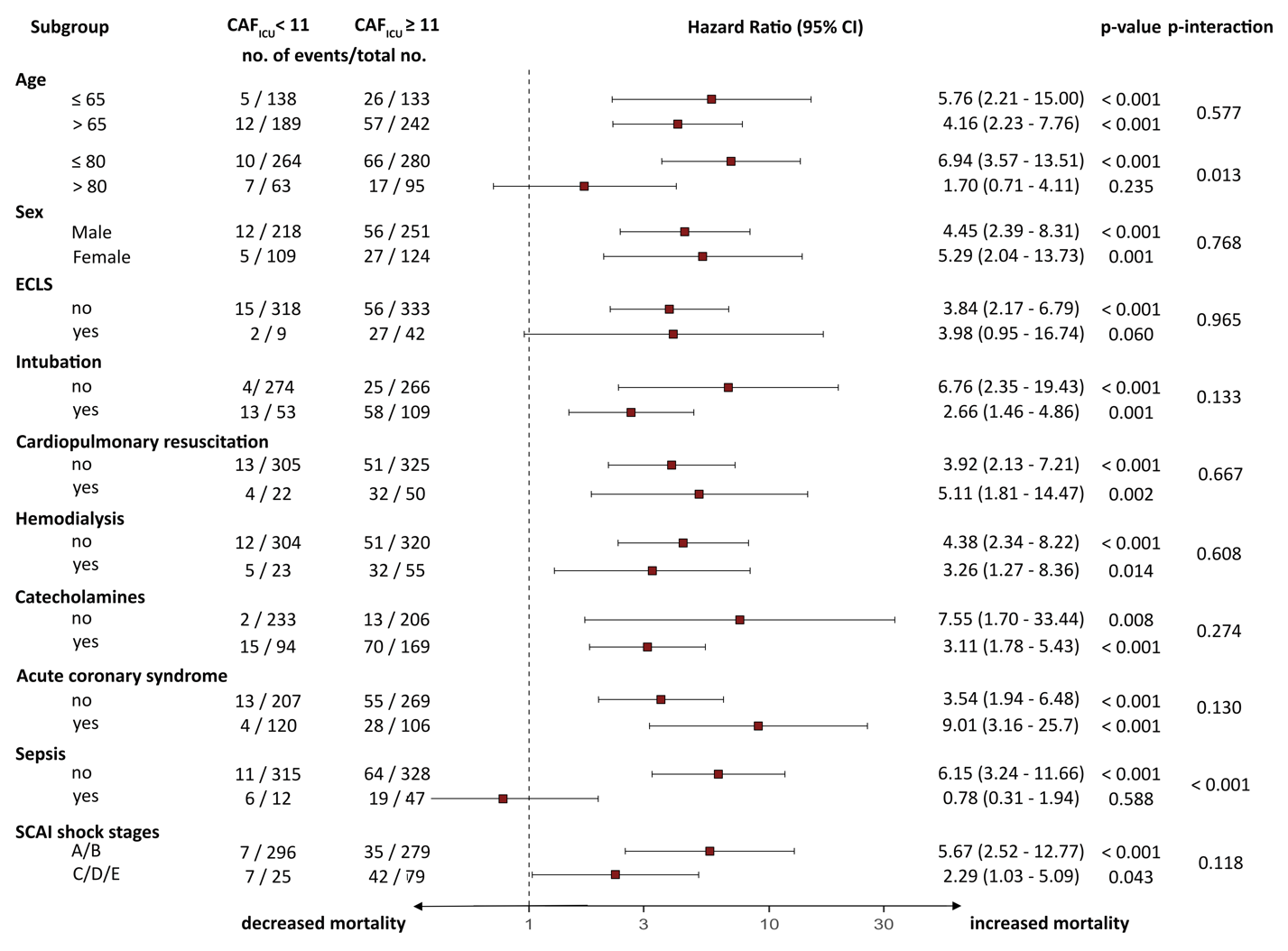
**

**Supplementary Figure 4** Subgroup analysis stratified by cardiac autonomic function score (CAF_ICU_) ≥ 11 units in the validation cohort. ECLS, extracorporeal life support

**
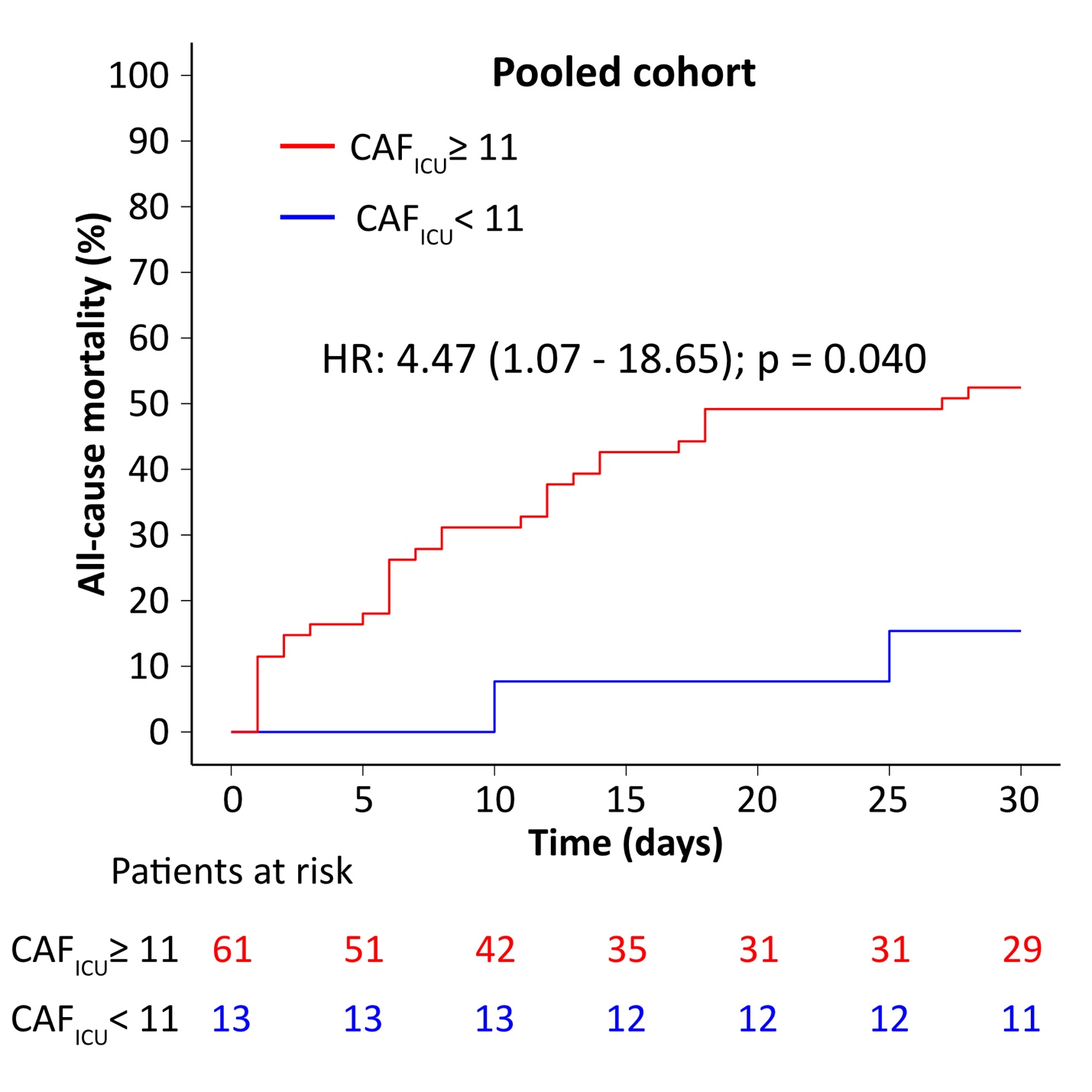
**

**Supplementary Figure 5** Cumulative mortality rates in patients treated with ECLS stratified by cardiac autonomic function score (CAF_ICU_) ≥ 11 units (red line) and < 11 units (blue line)

**
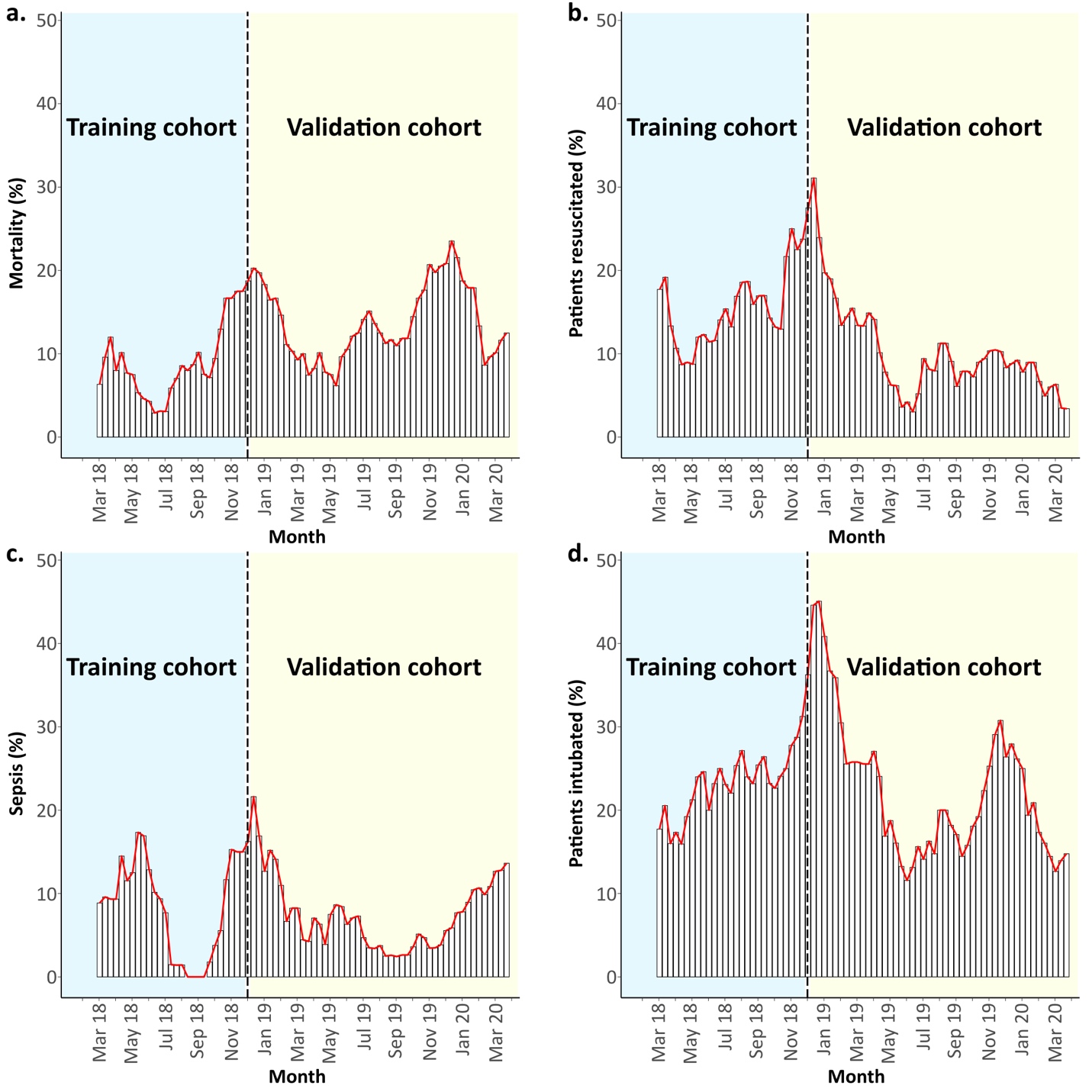
**

**Supplementary Figure 6** Mortality (a), cardiopulmonary resuscitation (b), intubation (c) and sepsis rates (d) over time in the training and validation cohorts. For the calculation of the rates a sliding window with size = 60 days and step size of 10 days was considered. The reported month is located in the middle of the sliding window
